# *Chiliadenus iphionoides* ethanolic extract suppresses hepatocellular carcinoma cell survival through ROS-associated mitochondrial dysfunction and intrinsic apoptotic signaling in p53-null Hep3B cells

**DOI:** 10.3389/fmed.2026.1914802

**Published:** 2026-09-10

**Authors:** Ahmad Salhab, Moshe Hodzaev HaCohen, Mutasem Natsheh, Adeeb Abu Sabalan, Ali Alayan, Ronit Ahdut HaCohen

**Affiliations:** 1 Department of Science, David Yellin Academic College of Education, Jerusalem, Israel; 2 Institute of Drug Research, School of Pharmacy, Faculty of Medicine, Hebrew University, Jerusalem, Israel; 3 Department of Education, Adam Mickiewicz University, Poznan, Poland

**Keywords:** apoptosis, *C. iphionoides*, cell-cycle modulation, hepatocellular carcinoma, natural products, oxidative stress, p53-independent signaling

## Abstract

**Background and aims:**

Hepatocellular carcinoma (HCC) is a cause of cancer-related mortality, and therapeutic efficacy is constrained by treatment resistance and toxicity. This study evaluated the anticancer activity and underlying molecular mechanisms of the *Chiliadenus iphionoides* ethanolic extract in hepatocellular carcinoma cells.

**Methods:**

Hep3B cells were treated with the *C. iphionoides* ethanolic extract (10–100 µg/mL) for 24 h. Cell viability, tumor-associated markers, intracellular reactive oxygen species (ROS), oxidative DNA damage, apoptosis, mitochondrial membrane potential, intrinsic apoptotic signaling, cell-cycle regulatory genes, and cell-cycle distribution were assessed using 3-(4,5-dimethylthiazol-2-yl)-5-(3-carboxymethoxyphenyl)-2-(4-sulfophenyl)-2H-tetrazolium (MTS), ELISA, RT-qPCR, fluorometric assays, and flow cytometry. ROS dependency was examined by N-acetyl-L-cysteine (NAC) pretreatment. Key findings were evaluated in HepG2 and Huh-7 HCC cells, and non-tumorigenic THLE-3 hepatocytes.

**Results:**

*C. iphionoides* reduced Hep3B viability in a concentration-dependent manner (IC50 = 35.2 µg/mL; 95% CI, 31.85–38.47) and suppressed the expression of alpha-fetoprotein, GPC-3, and VEGF-A. Treatment-induced ROS accumulation was accompanied by increased 8-hydroxy-2'-deoxyguanosine (8-OHdG) and *γ*H2AX levels, consistent with oxidative DNA damage. Mitochondrial depolarization was associated with Bax activation, increased caspase-9 activity, enhanced cleavage of caspase-3 and PARP-1, reduced BCL-2 expression, and increased Annexin V-positive apoptotic cells, indicating the activation of the intrinsic mitochondrial apoptotic pathway. Cyclin D1 mRNA was downregulated, whereas p21 mRNA increased despite the TP53-null background of Hep3B cells. Concordantly, G0/G1-phase accumulation increased from 50.1 ± 0.9% to 76.5 ± 1.4%, while S-phase cells decreased from 31.5 ± 0.7% to 11.2 ± 0.9%. NAC pretreatment substantially attenuated ROS accumulation, oxidative DNA damage, mitochondrial dysfunction, and apoptotic signaling, supporting an upstream role for ROS. Comparable responses were observed in HepG2 and Huh-7 cells, whereas THLE-3 hepatocytes were less affected, indicating the preferential rather than absolute sensitivity of malignant hepatocytes.

**Conclusion:**

*C. iphionoides* ethanolic extract exhibits anticancer activity in HCC cells *in vitro* through ROS-associated oxidative DNA damage, mitochondrial apoptosis, and p53-independent G0/G1 arrest.

## Introduction

Hepatocellular carcinoma (HCC) is one of the most lethal forms of cancer worldwide, contributing significantly to global morbidity and mortality (1). Its etiology is multifactorial, often linked to chronic liver conditions such as cirrhosis, hepatitis, and metabolic syndrome (2–4). Conventional therapeutic approaches, including surgical resection, loco-regional interventions, and systemic chemotherapy, are frequently compromised by drug resistance, tumor recurrence, and substantial adverse effects (5–7).

Natural products have long served as reservoirs of bioactive compounds with therapeutic potential in oncology (8, 9). Numerous plant-derived secondary metabolites, such as flavonoids and terpenoids, have been shown to possess antitumor properties, including antioxidative, anti-inflammatory, and pro-apoptotic activities (10–13). In particular, the genus *Chiliadenus (Asteraceae)* has a documented history in Mediterranean folk medicine as a source of bioactive constituents (14, 15). The comprehensive review by Castillo et al. collated evidence affirming the presence of an array of secondary metabolites, including flavonoids, terpenes, and essential oils, and their associated pharmacological activities that underpin its traditional use for anti-inflammatory, antioxidant, antimicrobial, and potential antitumor applications (14). Within the *Chiliadenus* genus, *Chiliadenus iphionoides* (*C. iphionoides*) has explicitly gained attention for its beneficial metabolic effects (15). Zandani et al. demonstrated that dietary supplementation of *C. iphionoides* in a mouse model of high-fat-diet-induced metabolic dysfunction-associated fatty liver disease significantly attenuated body weight gain, reduced hepatic steatosis, improved lipid metabolism, enhanced insulin sensitivity, and normalized liver enzyme levels (15). These findings suggest that it has hepatoprotective and metabolic-regulating properties, raising the prospect of its broader therapeutic implications in liver-related pathologies.

Although antitumor activity has been reported for *C. iphionoides* and related species (14), its mechanistic effects on hepatocellular carcinoma remain to be elucidated. In this study, we investigated the anticancer activity and underlying molecular mechanisms of the *C. iphionoides* ethanolic extract in Hep3B hepatocellular carcinoma cells, with a particular focus on reactive oxygen species (ROS)-associated oxidative DNA damage, mitochondrial apoptotic signaling, and p53-independent regulation of cell-cycle-associated genes. We hypothesized that the *C. iphionoides* ethanolic extract suppresses hepatocellular carcinoma cell survival by driving intracellular ROS accumulation, which in turn produces oxidative DNA damage, mitochondrial depolarization, and activation of the intrinsic apoptotic cascade, independent of p53. Therefore, the specific objectives were to determine the cytotoxic potency of the extract in Hep3B cells, define the upstream role of ROS using direct ROS quantification and antioxidant rescue, and establish whether the observed responses are reproducible in additional hepatocellular carcinoma cell lines while sparing non-tumorigenic hepatocytes.

## Materials and methods

### Collection, authentication, and preparation of the *C. iphionoides* ethanolic extract

Aerial parts of *C. iphionoides* (Boiss. & Blanche) Brullo were collected from the Jerusalem Hills, Israel, during the peak flowering season (April 2024), a period associated with elevated secondary metabolite accumulation in *C. iphionoides* and related Asteraceae species (13). As reported by Zandani et al., a voucher specimen of this species (No. 10 25 32) was deposited in the Herbarium of the National Natural History Collection, Hebrew University of Jerusalem (HUJI) (15). The plant material used in the present study was taxonomically authenticated against this reference material, and a voucher specimen of the collected batch (AS-2024-001) was deposited at the Department of Science, David Yellin Academic College of Education, Jerusalem, for future reference. The collection was conducted in compliance with the Israel Nature and Parks Authority regulations, and the geographic coordinates and collection date were recorded. Fresh plant material was rinsed with distilled water, air-dried in the dark at 20°C–25°C with forced ventilation to a constant weight, and milled to a fine powder (≤1 mm particle size; 18-mesh) using a stainless-steel mill. A total of 500 g of dried powdered aerial material was macerated in 5 L of absolute ethanol (plant-to-solvent ratio 1:10, w/v) at room temperature for 24 h with continuous magnetic stirring. The extraction was repeated three times using a fresh solvent to maximize metabolite recovery, and the combined extracts were filtered through Whatman No. 1 filter paper. The combined filtrates were concentrated under reduced pressure at ≤40°C using a rotary evaporator and further dried under vacuum to a constant weight, yielding 42.8 g of crude ethanolic extract (extraction yield 8.6% w/w relative to the dried plant material). The extracts were stored in amber glass vials at −20°C until use. For cell-based assays, the dried extract was dissolved in DMSO (100 mg/mL), sterile-filtered (0.22 µm PES membrane), and diluted in complete culture medium immediately before use. Final vehicle controls contained ≤01% (v/v) DMSO, a concentration that preliminary assays confirmed had no measurable effect on the cell viability. Three independent extraction batches were prepared using this standardized protocol and gave comparable extraction yields (8.6 ± 0.4%, mean ± SD) and comparable biological activity; one representative batch was used for all experiments reported here.

### Cell culture

Hep3B human HCC cells were cultured in RPMI-1640 medium (Biowest, Cat. No. MS027R100A) supplemented with 10% fetal bovine serum, 100 U/mL penicillin, 100 μg/mL streptomycin, and 1% L-glutamine. Cultures were maintained at 37°C in a humidified atmosphere containing 95% air and 5% CO_2_. The Hep3B cell line was routinely tested for mycoplasma contamination using PCR-based assays and was confirmed to be negative prior to experimentation. For treatments, Hep3B cells were seeded in six-well plates (1 × 10⁵ cells/well) and exposed to the *C. iphionoides* ethanolic extract at concentrations of 10, 25, 50, and 100 µg/mL for 24 h. In another set of experiments, Hep3B cells were pretreated with 5 mM N-acetyl-L-cysteine (NAC) for 1 h before exposure to *C. iphionoides* ethanolic extract (50 µg/mL). Following pretreatment, the medium was supplemented with *C. iphionoides* extract, and the cells were further incubated for 24 h in the presence of NAC. A concentration of 50 µg/mL was selected for the NAC rescue experiments because it induced robust oxidative stress, mitochondrial dysfunction, and apoptotic signaling while retaining a sufficiently viable cell population for evaluating pharmacological rescue; 100 µg/mL was not used because it caused near-maximal cytotoxicity, which would limit both the detection and interpretation of a protective effect. All NAC experiments included matched vehicle control, extract alone, NAC alone, and NAC-plus-extract groups generated within the same experimental series.

### MTS cell viability assay

Hep3B cells were seeded at a density of 1 × 10^3^ cells/well in 96-well flat-bottom plates and allowed to adhere overnight. The cells were then treated with *C. iphionoides* ethanolic extract (10, 25, 50, and 100 µg/mL) or vehicle control (≤0.1% DMSO) for 24 h in complete RPMI-1640 medium. Cell viability was quantified using an 3-(4,5-dimethylthiazol-2-yl)-5-(3-carboxymethoxyphenyl)-2-(4-sulfophenyl)-2H-tetrazolium (MTS) assay (Cat#197010; Abcam) according to the manufacturer's instructions. Briefly, MTS reagent was added to each well at 20% v/v (20 into 100 µL medium), and the plates were incubated for 2 h at 37°C in 5% CO_2_, protected from light. The absorbance was immediately measured at 490 nm using an ELISA reader (Tecan M100 Plate Reader). To control for any potential absorbance or chemical interference of the plant extract with the MTS reagent, extract-only blank wells containing complete culture medium, the corresponding concentration of *C. iphionoides* extract, and MTS reagent, but no cells, were included for all concentrations in each experiment. The absorbance of these extract-only blanks, along with that of the medium-only blanks, was subtracted from the corresponding experimental wells before viability was calculated as follows: % viability = [(A₄₉₀ sample−A₄₉₀ extract blank)/(A₄₉₀ vehicle−A₄₉₀ medium blank)] × 100. IC_50_ values were calculated from dose–response curves generated by non-linear regression using a four-parameter logistic (variable-slope) model using GraphPad Prism version 10.3.1 (GraphPad Software, San Diego, CA, USA), applied to normalized viability data from five technical replicates per concentration, and are reported along with the corresponding 95% confidence interval. The dose–response curve used for IC_50_ determination is shown in Supplementary Figure S1, and the underlying replicate values are provided in Supplementary Table S1.

### Quantification of HCC-associated markers by ELISA

Secreted alpha-fetoprotein (AFP) levels in the culture medium and Glypican-3 (GPC-3) concentrations in cell lysate were quantified using the Human Alpha-Fetoprotein Quantikine ELISA Kit (R&D Systems, DAFP00) and Human Glypican 3 ELISA Kit (Abcam; ab267640), respectively, according to the manufacturer's instructions. Briefly, following treatment, the cell culture media were collected, centrifuged at 1,000 × g for 10 min to remove debris, and stored at −20°C until analysis. Samples and standards were added to microplate wells precoated with a monoclonal antibody specific to human AFP or GPC-3, and the wells were incubated for a specified period. After the washing steps, enzyme-linked detection antibodies were applied, and colorimetric signals were developed using the supplied substrate solution. The absorbance was measured at 450 nm using a Tecan M100 microplate reader. Protein concentrations were calculated from standard curves generated using known concentrations of the recombinant standards. Secreted AFP was measured in a fixed volume of conditioned medium collected from each well and is reported as the absolute concentration recovered; therefore, it was not corrected for the number of cells remaining at the end of treatment. GPC-3 was measured in whole-cell lysates and normalized to the total protein concentration of the same lysate, determined in parallel using the Pierce BCA Protein Assay Kit (Thermo Scientific, Cat. No. 23227) and expressed as nanograms of GPC-3 per mg of lysate protein.

### Flow cytometry

Hep3B cells were seeded as described, and treated with *C. iphionoides* ethanolic extract (10, 25, 50, and 100 µg/mL) or vehicle control (≤0.1% DMSO) for 24 h. Both floating and adherent cells were collected by saving the culture supernatant, detaching adherent cells using an EDTA-free dissociation method, and combining the two fractions. Cells were washed twice with cold phosphate-buffered saline (PBS) at approximately 1 × 10⁶ cells/mL. For staining, 100 µL of cell suspension (∼1 × 10⁵ cells) was incubated with antibodies for activated conformational form of Bax (Cat# MA5-14003; Thermo Fisher Scientific), cleaved caspase-3 (Brilliant Violet 421, Cat# 570786; BD Biosciences), cleaved poly[ADP-ribose] polymerase 1 (PARP-1—AF647, Cat# ab237433; Abcam) and BCL-2 (AF488, Cat# ab692; Abcam). Bax was conjugated with secondary Goat Anti-Mouse IgG H&L (PE, Cat# ab97024; Abcam). Appropriate controls, including unstained, single-stained, and fluorescence-minus-one controls, were included for compensation and quadrant gating. Representative flow cytometry plots, along with the complete gating strategy applied to all markers, are provided in Supplementary File FCS dot blots. A minimum of 10,000 singlet events per sample was acquired. Cells were fixed and permeabilized using Cytofix/Cytoperm solution prior to intracellular staining. All stained cells were examined by flow cytometry with a BD LSR Fortessa Cell Analyzer (Becton Dickinson Immunofluorometry Systems) and analyzed using FCS Express 7 (De Novo Software).

### Intracellular ROS measurements by DCFH-DA flow cytometry

Intracellular ROS were quantified using the cell-permeable fluorescent probe 2′,7′-dichlorodihydrofluorescein diacetate (DCFH-DA). Hep3B cells were treated with *C. iphionoides* ethanolic extract (10, 25, 50, and 100 µg/mL) or vehicle control (≤0.1% DMSO) for 24 h. Following treatment, the cells were harvested, washed twice with PBS, and incubated with 10 µM DCFH-DA in serum-free medium for 30 min at 37°C in the dark. Cells were then washed twice with PBS to remove excess probe and analyzed immediately using a BD LSRFortessa flow cytometer. Dichlorofluorescein-'7,'2 (DCF) fluorescence was detected using the 488-nm excitation laser and the FITC channel (530/30 nm emission filter), and the data are reported as the mean fluorescence intensity (MFI) of DCF, analyzed using FCS Express 7.0 software. The same procedure was followed for the NAC rescue experiments.

### Annexin V-FITC/propidium iodide apoptosis assay

Apoptotic cell death was assessed using the Annexin V-FITC/propidium iodide (PI) Apoptosis Detection Kit (BD Biosciences) according to the manufacturer's instructions. Briefly, unfixed cells were resuspended in 1  ×   binding buffer, and 100 µL of the cell suspension (approximately 1 × 10⁵ cells) was incubated with 5 µL Annexin V-FITC and 5 µL PI for 15 min at room temperature in the dark. After incubation, 400 µL of binding buffer was added, and the samples were analyzed immediately by flow cytometry. Cell populations were classified as viable (Annexin V⁻/PI⁻), early apoptotic (Annexin V⁺/PI⁻), late apoptotic or secondary necrotic (Annexin V⁺/PI⁺), and necrotic (Annexin V⁻/PI⁺).

### Cell-cycle analysis using DNA content flow cytometry

Cell-cycle distribution was determined by PI DNA content flow cytometry. Hep3B cells were treated with *C. iphionoides* ethanolic extract (10, 25, 50, and 100 µg/mL) or vehicle control (≤0.1% DMSO) for 24 h. Both floating and adherent cells were collected, washed twice with cold PBS, fixed in ice-cold 70% ethanol added dropwise under gentle vortexing, and stored at −20°C until analysis. The fixed cells were washed, treated with RNase A to eliminate double-stranded RNA, and stained with PI. DNA content was determined using a BD LSRFortessa flow cytometer; doublets were excluded based on forward-scatter area versus height, and a minimum of 10,000 single-cell events was collected per sample. Sub-G1 events were excluded from the analysis; therefore, the reported proportions of cells in G0/G1, S, and G2/M refer to the gated cycling population and are independent of the apoptotic fraction quantified separately by Annexin V/PI staining. Data were analyzed using FCS Express 7 (De Novo Software) with three replicates per treatment group. Because the three phase proportions are compositional and sum to 100% within each sample, treatment effects were evaluated within each phase separately using Tukey's multiple comparison test rather than by interpreting an overall treatment main effect.

### Validation in additional hepatocellular carcinoma and non-tumorigenic hepatocyte cell lines

To determine whether the observed responses were reproducible beyond Hep3B cells and whether they were selective for malignant hepatocytes, two additional hepatocellular carcinoma cell lines (HepG2 and Huh-7) and non-tumorigenic THLE-3 hepatocytes were treated with *C. iphionoides* ethanolic extract (50 µg/mL) or vehicle control (≤0.1% DMSO) for 24 h. *MKI67* and *VEGF-A* mRNA expression were determined by RT-qPCR and normalized to *GAPDH*. *γ*H2AX positivity and mitochondrial membrane potential (JC-1 assay) were assessed by flow cytometry, and caspase-9 activity was measured by fluorometric assay and normalized to total protein concentration, exactly as described above for Hep3B cells. The validation experiments were performed in triplicate for each group.

### Caspase-9 activity assay

Caspase-9 activity was quantified using Abcam's Caspase-9 Assay Kit (Fluorometric) (Abcam, Cat# ab65607), according to the manufacturer's instructions. Briefly, Hep3B cells were treated with *C. iphionoides* ethanolic extract (10, 25, 50, and 100 µg/mL) for 24 h, harvested, washed twice with cold PBS, and lysed in chilled cell lysis buffer supplied with the kit. Cell lysates were incubated on ice for 10 min and centrifuged at 10,000 × g for 1 min to collect the supernatant. The total protein concentration in each lysate was determined using the Pierce™ BCA Protein Assay Kit (Thermo Scientific, Cat. No. 23227) according to the manufacturer's instructions. Briefly, aliquots of each lysate were mixed with the BCA working reagent and incubated at 37°C for 30 min, absorbance was measured at 562 nm using a Tecan M100 microplate reader, and protein concentrations were calculated from a bovine serum albumin standard curve generated in parallel with the samples. Equal amounts of total protein from each sample were incubated with the caspase-9-specific fluorogenic substrate LEHD-AFC in reaction buffer containing Dithiothreitol (DTT) for 2 h at 37°C in the dark. Cleavage of LEHD-AFC by active caspase-9 releases free AFC, generating yellow-green fluorescence. Fluorescence intensity was measured using a fluorescence microplate reader at excitation/emission wavelengths of 400/505 nm. Caspase-9 activity was normalized to the total protein concentration and expressed as relative fluorescence units (RFU) relative to untreated control cells. Data are presented as mean ± SD of five technical replicates.

### Assessment of oxidative DNA damage using 8-OHdG ELISA

Intracellular oxidative DNA damage was quantified by measuring 8-hydroxy-2'-deoxyguanosine (8-OHdG) levels using a competitive ELISA kit (Cat# 285254; Abcam, Cambridge, UK), according to the manufacturer's protocol. Briefly, Hep3B cells were treated with *C. iphionoides* ethanolic extract (10, 25, 50, or 100 µg/mL) or vehicle control for 24 h, harvested, washed twice with cold PBS, and lysed using the DNA extraction buffer provided in the kit. Genomic DNA was extracted, enzymatically hydrolyzed to nucleosides, and subjected to a competitive immunoassay in which sample 8-OHdG competed with an 8-OHdG-acetylcholinesterase conjugate for antibody binding sites on precoated microplate wells. After washing, Ellman's reagent was added, and the absorbance was measured at 412 nm (Tecan M100 Plate Reader). 8-OHdG levels were determined from a standard curve of known concentrations and expressed as ng 8-OHdG per 10^6^ Hep3B cells. To account for treatment-induced differences in cell numbers, viable cells were counted using a Neubauer hemocytometer following trypan blue exclusion. Cell suspensions were mixed with 0.4% trypan blue solution in a 1:1 ratio, and viable (unstained) cells were counted manually under a light microscope according to standard hemocytometer procedures. The resulting counts were used to normalize the measured 8-OHdG concentrations, which were expressed as ng of 8-OHdG per 10⁶ viable cells.

### Mitochondrial membrane potential assessment

Mitochondrial membrane potential (*Δψ*m) was assessed using JC-1 Dye (Cat# T3168, Thermo Fisher), followed by flow cytometric analysis. Hep3B cells were seeded and treated with *C. iphionoides* ethanolic extract at concentrations of 10, 25, 50, and 100 µg/mL for 24 h. Following treatment, both floating and adherent cells were collected, washed twice with PBS, and incubated with JC-1 staining solution for 20 min at 37°C in the dark, according to the manufacturer's instructions. Both floating and adherent cells were collected because apoptotic cells frequently lose adherence and detach from the culture surface during treatment. Excluding the floating fraction preferentially omits apoptotic cells undergoing mitochondrial depolarization and therefore underestimates the extent of mitochondrial dysfunction. Inclusion of both fractions minimizes sampling bias and provides an unbiased assessment across the entire cell population.

After staining, the cells were washed with assay buffer and immediately analyzed by flow cytometry. JC-1 aggregates, reflecting preserved mitochondrial membrane potential, were detected in the red fluorescence channel, whereas JC-1 monomers, reflecting mitochondrial depolarization, were detected in the green fluorescence channel. Mitochondrial polarization was quantified as the percentage of cells exhibiting predominant red JC-1 aggregate fluorescence. A minimum of 10,000 singlet events were acquired per sample after the exclusion of debris and doublets.

### RNA isolation, cDNA synthesis, and quantitative real-time PCR

Total cellular RNA was isolated from treated and vehicle-control Hep3B cells using TRI Reagent, according to the manufacturer's instructions. Cells were lysed directly in TRI Reagent and homogenized thoroughly. Chloroform was added, and the samples were mixed vigorously, incubated at room temperature, and centrifuged at 12,000 × g for 15 min at 4°C to achieve phase separation. The upper aqueous phase containing the RNA was carefully transferred to a fresh RNase-free microcentrifuge tube. For RNA precipitation, 0.5 mL of isopropanol (Bio Lab; Cat# 16260521) was added, the samples were incubated at 25°C for 10 min, and centrifuged at 12,000 × g for 10 min at 4°C. The supernatants were removed, and 1 mL of 75% ethanol was added to the pellets before centrifugation at 7,500 × g for 5 min at 4°C. The pellets were air-dried at room temperature for 15 min, 50 μL of Diethyl dicarbonate (DEPC)-treated water was added, and the samples were incubated for 10 min at 55°C. To minimize genomic DNA contamination, the isolated RNA was treated with RNase-free DNase I according to the manufacturer's instructions, followed by enzyme inactivation, as recommended by the supplier. RNA concentration and purity were assessed using a NanoDrop ND-1000 spectrophotometer, and only samples with an A260/A280 ratio of approximately 1.8–2.0 (mean=1.98) were used for downstream analysis. Equal amounts of total RNA (500 ng) were reverse-transcribed using a High-Capacity cDNA Reverse Transcription Kit (R&D; Cat# 1406197). Quantitative real-time PCR (RT-qPCR) was performed to quantify *VEGF-A*, *cyclin D1*, and *p21* gene expression using commercially predesigned TaqMan™ Gene Expression Assays (Thermo Fisher Scientific, Waltham, MA, USA) and TaqMan Fast Advanced Master Mix (Cat# 4371130, Applied Biosystems). The primer and probe sequences of these predesigned assays are proprietary and are not publicly released by the manufacturer; therefore, the corresponding assay identification numbers are given here to allow full reproducibility: *VEGF-A* (Hs00242628_m1), *CCND1* (cyclin D1; Hs00183683_m1), *CDKN1A* (p21; Hs01075288_m1), *MKI67* (Hs06597542_g1), and *GAPDH* (Hs99999905_m1). Gene expression was normalized to that of the housekeeping gene *GAPDH*. The cycling conditions consisted of initial denaturation at 95°C for 20 s, followed by 40 cycles at 95°C for 1 s and 60°C for 20 s. The data were analyzed using a QuantStudio™ 5 Real-Time PCR System (Cat# A34322, Applied Biosystems). Relative gene expression was calculated using the comparative Ct (2−*ΔΔ*Ct) method, and equal amounts of total RNA were used for cDNA synthesis in all the experimental groups. GAPDH was used as the sole reference gene in the present study; its expression stability was not independently verified against additional housekeeping genes, which is acknowledged as a limitation in the Discussion section.

### Statistical analysis

All statistical analyses were performed using GraphPad Prism version 10.3.1 (GraphPad Software, San Diego, CA, USA). For experiments involving a single independent variable, differences among groups were analyzed using one-way ANOVA, followed by Tukey's multiple comparisons test. NAC rescue experiments, which involved two independent factors (extract treatment and NAC pretreatment), were analyzed using two-way ANOVA, followed by Šídák's multiple comparisons test. A *p*-value ≤ 0.05 was considered statistically significant. Unless otherwise stated, results are presented as mean ± SD from five technical replicates (*n* = 5 per group) performed within each experiment; the validation experiments in HepG2, Huh-7, and THLE-3 cells were performed with three replicates per group. The graphical abstract was created using FigureLabs.

## Results

### *C. iphionoides* reduces Hep3B cell viability and tumor-marker expression

To determine whether the *C. iphionoides* ethanolic extract reduced the viability of Hep3B cells, cell viability was evaluated using the MTS assay. Hep3B cells were treated with increasing concentrations of the extract (10–100 µg/mL) for 24 h. A modest reduction in viability was observed at 10 µg/mL (92.1 ± 3.0% of control), while progressively greater inhibition occurred at higher concentrations, with viability decreasing to 67.1 ± 2.8%, 35.8 ± 2.1%, and 14.7 ± 1.9% at 25, 50, and 100 µg/mL, respectively. Non-linear regression using a four-parameter logistic variable-slope model yielded an IC_50_ of 35.2 µg/mL (95% CI: 31.85–38.47 µg/mL), with a Hill slope of 1.93 and a goodness-of-fit of *R*^2^ = 0.9953. The analysis included five extract concentrations, each with five technical replicates, for a total of 25 observations. The corresponding dose–response curve and the underlying replicate values are provided in Supplementary Figure S1 and Supplementary Table S1, respectively. These findings demonstrate a clear, concentration-dependent reduction in Hep3B cell viability *in vitro* and provide a basis for the subsequent mechanistic experiments. The absorbance readings were corrected against extract-only blank wells, excluding the contribution of the extract itself to the measured signal. Because tetrazolium-based assays report metabolic activity rather than cell number, the term viability is used here in that sense, and *C. iphionoides* ethanolic extract is referred to as reducing cell viability rather than being directly cytotoxic (Table 1).

**Table 1:** *Chiliadenus iphionoides* ethanolic extract suppresses Hep3B cell viability in a concentration-dependent manner.

Concentration (µg/mL)	Cell viability (% of control) mean ± SD	Reduction vs. control (%)
0 (vehicle)	100.0 ± 2.5	—
10	92.1 ± 3.0	7.9
25	67.1 ± 2.8*	32.9
50	35.8 ± 2.1*	64.2
100	14.7 ± 1.9*	85.3

Hep3B cells were treated for 24 h with *C. iphionoides* ethanolic extract or vehicle control (0.1% DMSO). Viability was determined by the MTS assay; absorbance at 490 nm was background-corrected against both medium-only and extract-only blank wells (medium + extract + MTS reagent, without cells) and expressed relative to vehicle-treated cells, normalized to 100%. Data are mean ± SD of five technical replicates (*n* = 5 per concentration); individual replicate values are given in Supplementary Table S1. Statistical significance was determined by one-way ANOVA followed by Tukey's multiple comparisons test. Asterisks denote comparisons with the vehicle-treated control group (*p < 0.05). The IC_50_ was 35.2 µg/mL (95% CI 31.85–38.47 µg/mL; Hill slope 1.93; *R*^2^ = 0.9953), calculated by non-linear regression using a four-parameter logistic (variable-slope) model in GraphPad Prism version 10.3.1. The dose–response curve is shown in Supplementary Figure S1.

**Figure 1:**
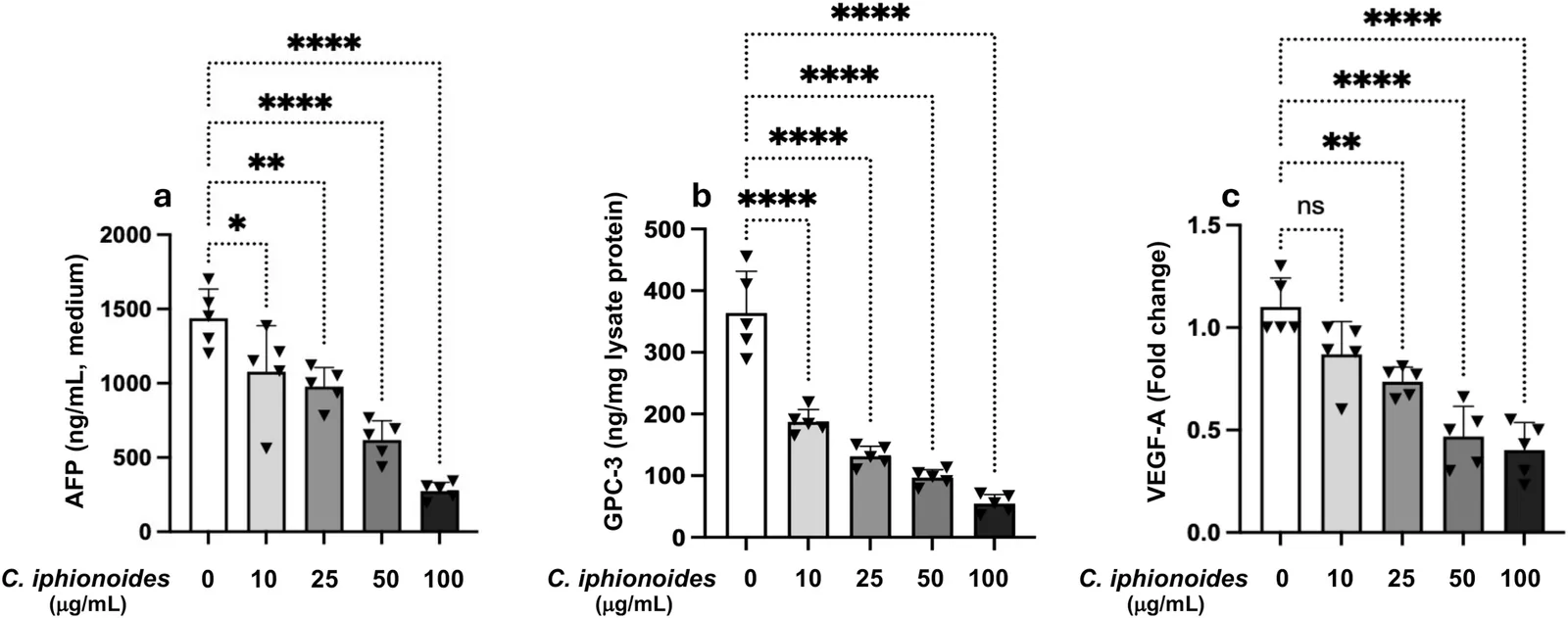
*C. iphionoides* decreases Hep3B cell activity. Hep3B cells were incubated with increasing concentrations of the ethanolic extract (10, 25, 50, and 100 µg/mL) or vehicle control (0.1% DMSO) for 24 h. **(a)** Secreted alpha-fetoprotein (AFP) in the conditioned medium. **(b)** Glypican-3 (GPC-3) in cell lysates was quantified by ELISA. AFP was expressed as the absolute concentration recovered per well (ng/mL) and was not corrected for cell numbers; GPC-3 was normalized to the total protein concentration of the same lysate and expressed as ng/mg lysate protein. **(c)** *VEGF-A* mRNA expression was determined by RT-qPCR, normalized to GAPDH, and expressed as the fold change relative to the vehicle-treated control group. Data are presented as mean ± SD of five technical replicates. Statistical significance was determined using one-way ANOVA followed by Tukey's multiple comparisons test. Asterisks indicate comparisons with the vehicle-treated control group (0.1% DMSO): *p < 0.05, **p < 0.01, ***p < 0.001, ****p < 0.0001, ns; not significant.

AFP, GPC-3, and *VEGF-A* are well-established biomarkers of HCC that reflect both tumor cell activity and viability (16–18). Measuring the expression or secretion of these markers provides a functional readout complementary to direct cell viability assays, enabling the assessment of the impact of *C. iphionoides* extract on tumor-associated protein and transcript production. As shown in Figure 1a, treatment of Hep3B cells with *C. iphionoides* ethanolic extract for 24 h resulted in a concentration-dependent decrease in secreted AFP protein levels, as measured by ELISA in the conditioned medium. Untreated cells secreted an average of 1,438.0 ± 196.77 ng/mL AFP. Treatment with extracts at 10, 25, 50, and 100 µg/mL resulted in mean AFP concentrations of 1,077.2 ± 310.56, 977.0 ± 129.43, 617.4 ± 129.85, and 274.4 ± 57.71 ng/mL, respectively. This corresponded to a progressive, dose-dependent reduction in AFP secretion, with decreases of approximately 25%, 32%, 57%, and 81% relative to the untreated controls (Figure 1a). Moreover, quantitative analysis of GPC-3 protein levels in Hep3B cell lysates by ELISA revealed a progressive and dose-dependent reduction following treatment. In untreated control cells, the mean GPC-3 expression level was 364.0 ± 64.4 ng/mg protein isolated from the cells. Treatment with 10, 25, 50, and 100 µg/mL reduced GPC-3 to 187.4 ± 17.3, 131.6 ± 15.1, 97.0 ± 13.2, and 54.6 ± 14.0 ng/mg lysate protein, respectively, corresponding to reductions of approximately 49%, 64%, 73%, and 85% relative to untreated controls (Figure 1b). Consistent with the AFP and GPC-3 trends, *VEGF-A* mRNA levels, determined by RT-qPCR and normalized to GAPDH, decreased significantly with increasing *C. iphionoides* concentrations (Figure 1c). Mean relative mRNA expression was 1.10 ± 0.13 in untreated cells, 0.87 ± 0.15 at 10 μg/mL, 0.74 ± 0.07 at 25 μg/mL, 0.47 ± 0.14 at 50 μg/mL, and 0.40 ± 0.12 at 100 μg/mL (*p* < 0.01). The suppression became prominent at ≥25 μg/mL, corresponding to approximately 33%–64% inhibition compared to the control. Taken together, these data support the hypothesis that *C. iphionoides* ethanolic extract reduces hepatocellular carcinoma cell viability and the expression of tumor-associated markers *in vitro*, providing a basis for further mechanistic experiments. Because secreted AFP is reported as an absolute concentration, its reduction reflects the combined contribution of a smaller viable cell population and any change in secretion per surviving cell and should be interpreted accordingly. In contrast, GPC-3, normalized to lysate protein, and *VEGF-A,* normalized to *GAPDH* with equal RNA input, were both independent of cell numbers and both decreased markedly across the same concentration range. Therefore, the suppression of tumor-associated marker expression was not solely attributed to the lower cell numbers in the treated wells.

### *C. iphionoides* induces ROS-associated oxidative DNA damage

**Figure 2:**
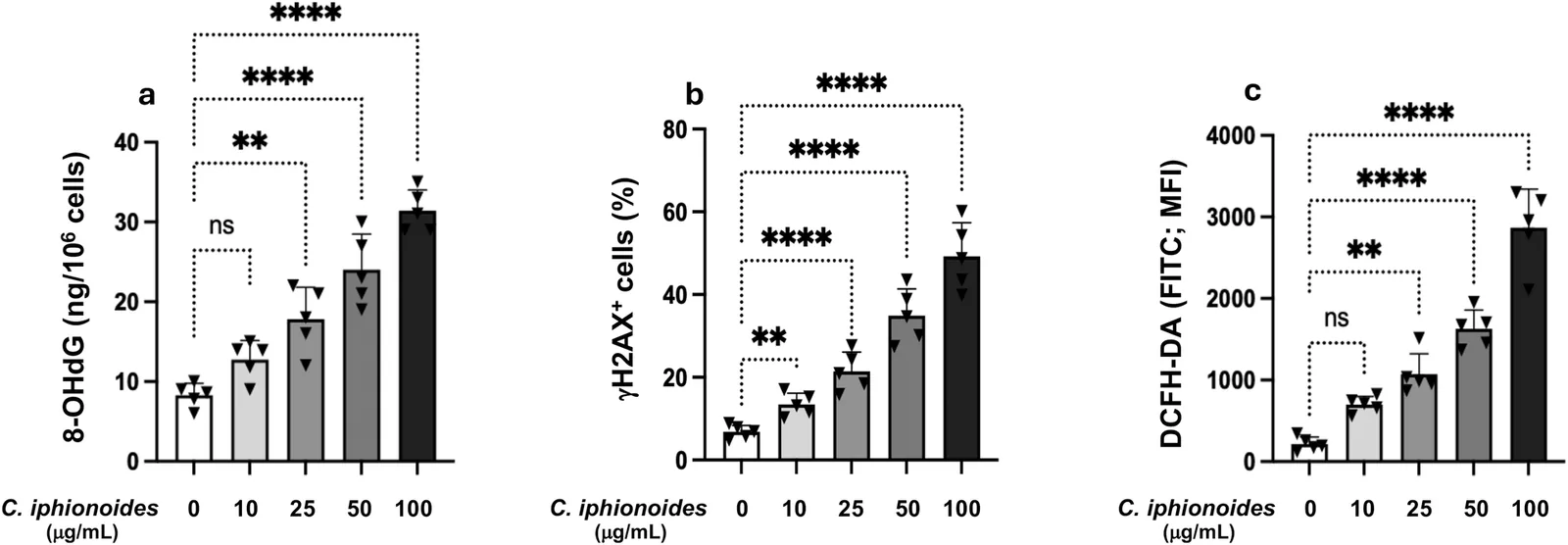
*C. iphionoides* ethanolic extract induces intracellular ROS accumulation and oxidative DNA damage in Hep3B cells. Hep3B cells were treated with increasing concentrations of *C. iphionoides* ethanolic extract (10, 25, 50, and 100 µg/mL) for 24 h. **(a)** Intracellular 8-hydroxy-2′-deoxyguanosine (8-OHdG) levels were quantified by competitive ELISA and expressed as ng/10⁶ cells. **(b)** *γ*H2AX-positive cells were quantified by flow cytometry and expressed as the percentage of *γ*H2AX-positive cells. **(c)** Intracellular reactive oxygen species (ROS) generation was assessed using the 2′,7′-dichlorodihydrofluorescein diacetate (DCFH-DA) assay. Following DCFH-DA loading, DCF fluorescence was quantified by flow cytometry in the FITC channel and expressed as the mean fluorescence intensity (MFI). Data are presented as mean ± SD of five technical replicates. Statistical significance was determined using one-way ANOVA followed by Tukey's multiple comparisons test. Asterisks indicate comparisons with the vehicle-treated control group (0.1% DMSO): **p < 0.01, ****p < 0.0001, ns; not significant.

ROS can induce oxidative modifications in DNA, leading to strand breaks, genomic instability, and activation of stress-response pathways (19). One of the most prevalent and stable markers of ROS-associated oxidative DNA injury is 8-hydroxy-2′-deoxyguanosine (8-OHdG), whereas the phosphorylation of histone H2AX (*γ*H2AX) is a sensitive marker of DNA double-strand breaks and DNA damage signaling (20–22). To determine whether *C. iphionoides* ethanolic extract induced oxidative stress and oxidative DNA damage in Hep3B cells, intracellular ROS generation, intracellular 8-OHdG levels, and *γ*H2AX expression were evaluated after 24 h of treatment. Untreated control cells exhibited 8-OHdG levels of 8.28 ± 1.50 ng/10⁶ Hep3B cells. Treatment with 10, 25, 50, and 100 µg/mL extract resulted in mean values of 12.74 ± 2.40, 17.80 ± 4.02, 24.00 ± 4.47, and 31.40 ± 2.61 ng/10⁶ cells, corresponding to increases of approximately 53.9%, 115.0%, 189.9%, and 279.2% relative to that of the control, respectively (Figure 2a). These results demonstrate a clear concentration-dependent elevation of oxidative DNA damage following treatment with the *C. iphionoides* extract.

To further confirm ROS-associated genotoxic stress, *γ*H2AX expression was quantified by flow cytometry. Untreated control cells demonstrated *γ*H2AX positivity of 6.8%. Treatment with *C. iphionoides* ethanolic extract at 10, 25, 50, and 100 µg/mL increased *γ*H2AX-positive Hep3B cells to 13.5 ± 2.1 (*p* < 0.01), 21.5 ± 3.6, 35 ± 5, and 49 ± 5.1%, respectively (*p* < 0.0001; Figure 2b). The marked concentration-dependent increase in *γ*H2AX expression indicates the activation of DNA damage signaling pathways and the accumulation of DNA double-strand breaks following treatment. Notably, the increase in *γ*H2AX paralleled the elevation in intracellular 8-OHdG levels, strongly supporting the involvement of ROS-mediated genotoxic stress in response to *C. iphionoides* treatment.

To directly determine whether the extract induced oxidative stress rather than only its downstream consequences, intracellular ROS levels were quantified using DCFH-DA and flow cytometry. As shown in Figure 2c, *C. iphionoides* induced a marked concentration-dependent increase in intracellular ROS levels. The basal ROS production in the untreated cells corresponded to a DCF fluorescence of 223.0 ± 74.7 MFI, whereas treatment with 10, 25, 50, and 100 μg/mL extract increased the DCF fluorescence to 699.0 ± 95.4, 1,070.0 ± 256.8, 1,628.4 ± 241.7, and 2,868.4 ± 507.8 MFI, respectively. These values corresponded to 3.24-, 4.95-, 7.54-, and 13.28-fold increases relative to untreated cells, demonstrating a robust dose-dependent induction of oxidative stress that preceded and accompanied the oxidative DNA damage described above.

### *C. iphionoides* induces mitochondrial dysfunction and activates intrinsic apoptotic signaling in Hep3B cells

**Figure 3:**
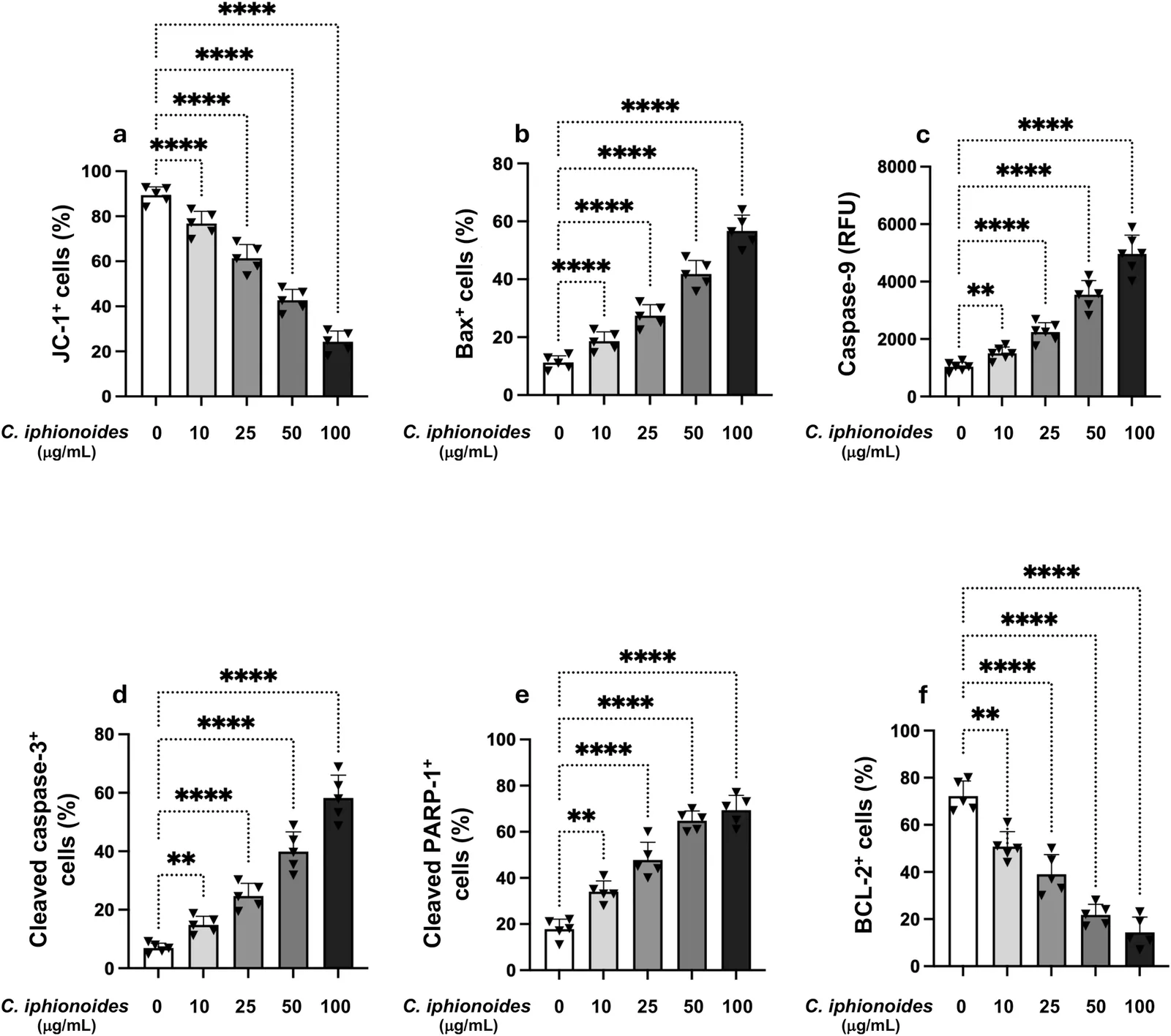
*C. iphionoides* ethanolic extract induces mitochondrial dysfunction and activates intrinsic apoptotic signaling in Hep3B cells. Hep3B cells were treated with increasing concentrations of *C. iphionoides* ethanolic extract (10, 25, 50, and 100 µg/mL) for 24 h. **(a)** Mitochondrial membrane potential was assessed using the JC-1 assay and expressed as a percentage of JC-1 aggregate-positive cells, as determined by flow cytometry. **(b)** Activated Bax expression, **(c)** caspase-9 activity, **(d)** cleaved caspase-3 expression, **(e)** cleaved PARP-1 levels, and **(f)** BCL-2 expression were quantified following treatment. Activated Bax, cleaved caspase-3, cleaved PARP-1, and BCL-2 were analyzed by flow cytometry and expressed as a percentage of positive cells, whereas caspase-9 activity was measured using a fluorometric assay and expressed as relative fluorescence units (RFU). Data are presented as mean ± SD of five technical replicates. Statistical significance was determined using one-way ANOVA followed by Tukey's multiple comparisons test. Asterisks indicate comparisons with the vehicle-treated control group (0.1% DMSO): **p < 0.01, ****p < 0.0001.

As excessive ROS-mediated DNA damage can disrupt mitochondrial integrity and trigger intrinsic apoptotic signaling (23, 24), the mitochondrial membrane potential (*ΔΨ*m) was evaluated using the JC-1 assay following treatment with *C. iphionoides* ethanolic extract for 24 h. Untreated Hep3B cells predominantly exhibited polarized mitochondria, with 89.4 ± 4.6% of JC-1 aggregate-positive cells. Treatment with 10, 25, 50, and 100 µg/mL extract progressively reduced mitochondrial polarization to 76.8 ± 5.2%, 61.4 ± 6.1%, 42.7 ± 5.4%, and 24.3 ± 4.8%, respectively (*p* < 0.0001) (Figure 3a). These findings indicate a marked concentration-dependent loss of the mitochondrial membrane potential and progressive mitochondrial depolarization in response to C. iphionoides treatment.

To further characterize the activation of the intrinsic apoptotic pathway, the activated conformational form of Bax, caspase-9 activity, cleaved caspase-3, cleaved PARP-1, and BCL-2 expression were evaluated after 24 h of treatment. Treatment with the *C. iphionoides* extract resulted in a marked concentration-dependent increase in Bax expression, increasing from 11.2 ± 2.3% in untreated control cells to 18.6 ± 3.0%, 27.4 ± 3.8%, 41.8 ± 4.6%, and 56.7 ± 5.2% following treatment with 10, 25, 50, and 100 µg/mL extract, respectively (*p* < 0.0001) (Figure 3b). Similarly, caspase-9 activity increased from 1,036 ± 177 RFU in untreated Hep3B cells to 1,502 ± 247, 2,244 ± 359, 3,542 ± 556, and 4,965 ± 736 RFU following treatment with 10, 25, 50, and 100 µg/mL extract, respectively (*p* < 0.0001) (Figure 3c). Moreover, cleaved caspase-3 expression demonstrated a parallel increase, increasing from 6.9 ± 1.5% in untreated controls to 14.8 ± 2.5%, 24.7 ± 3.4%, 39.9 ± 4.7%, and 58.2 ± 6.1% at increasing extract concentrations (*p* < 0.0001) (Figure 3d), indicating activation of the mitochondrial apoptotic cascade.

Consistent with the activation of apoptotic signaling, cleaved PARP-1 levels increased markedly following treatment. Cleavage of PARP-1 from its full-length 116-kDa form to the 89-kDa fragment is a well-recognized hallmark of irreversible apoptosis and caspase-mediated DNA damage (25). As shown in Figure 3e, treatment with C. iphionoides ethanolic extract for 24 h led to a significant concentration-dependent increase in cleaved PARP-1 levels, increasing from 17.8 ± 4.3% of cleaved PARP-1-positive cells in untreated controls to 34.0 ± 4.9%, 47.8 ± 7.2%, 64.8 ± 4.6%, and 69.4 ± 6.3% following treatment with 10, 25, 50, and 100 µg/mL extract, respectively (*p* < 0.0001), corresponding to an approximate 2- to 4-fold increase relative to untreated controls.

To further confirm the activation of intrinsic apoptotic signaling, BCL-2 expression was quantified in Hep3B cells following treatment with C. iphionoides extract. BCL-2 is a major antiapoptotic protein that preserves mitochondrial membrane integrity and inhibits cytochrome-c release, thereby preventing caspase activation (26–28). Suppression of BCL-2 expression, therefore, reflects a shift toward apoptosis (28). Treatment with increasing concentrations of C. iphionoides extract resulted in a pronounced concentration-dependent reduction in BCL-2 expression (Figure 3f). The percentage of BCL-2-positive Hep3B cells decreased progressively from 72.2 ± 6.1% in untreated controls to 50.8 ± 6.1%, 39.0 ± 8.5%, 21.8 ± 4.7%, and 14.4 ± 6.0% following treatment with 10, 25, 50, and 100 µg/mL extract, respectively (*p* < 0.0001). These reductions corresponded to decreases of approximately 30%, 46%, 70%, and 80% relative to untreated controls.

To confirm directly that the loss of viability reflects apoptotic rather than non-specific cell death, Annexin V-FITC/PI staining was performed. Treatment with *C. iphionoides* resulted in a concentration-dependent increase in the proportion of Annexin V⁺/PI⁻ Hep3B cells (Supplementary Figure S2). The percentage of early apoptotic cells increased from 12.0 ± 2.2% in untreated controls to 17.4 ± 2.3%, 27.4 ± 3.5%, 35.8 ± 8.1%, and 55.0 ± 9.0% following treatment with 10, 25, 50, and 100 μg/mL, respectively. The highest concentration produced an approximately 4.6-fold increase in early apoptotic cells relative to untreated controls, corroborating the activation of the intrinsic apoptotic pathway, as indicated by mitochondrial depolarization, caspase-9 activation, and cleaved caspase-3 induction.

Collectively, these findings demonstrate that the *C. iphionoides* ethanolic extract induces mitochondrial dysfunction and activates intrinsic apoptotic signaling in Hep3B cells through a ROS-associated mechanism characterized by Bax induction, mitochondrial depolarization, caspase activation, PARP-1 cleavage, suppression of BCL-2, and a concentration-dependent increase in Annexin V-positive apoptotic cells.

### *C. iphionoides* induces p53-independent G0/G1 arrest and regulation of cell-cycle-associated genes in Hep3B cells

**Figure 4:**
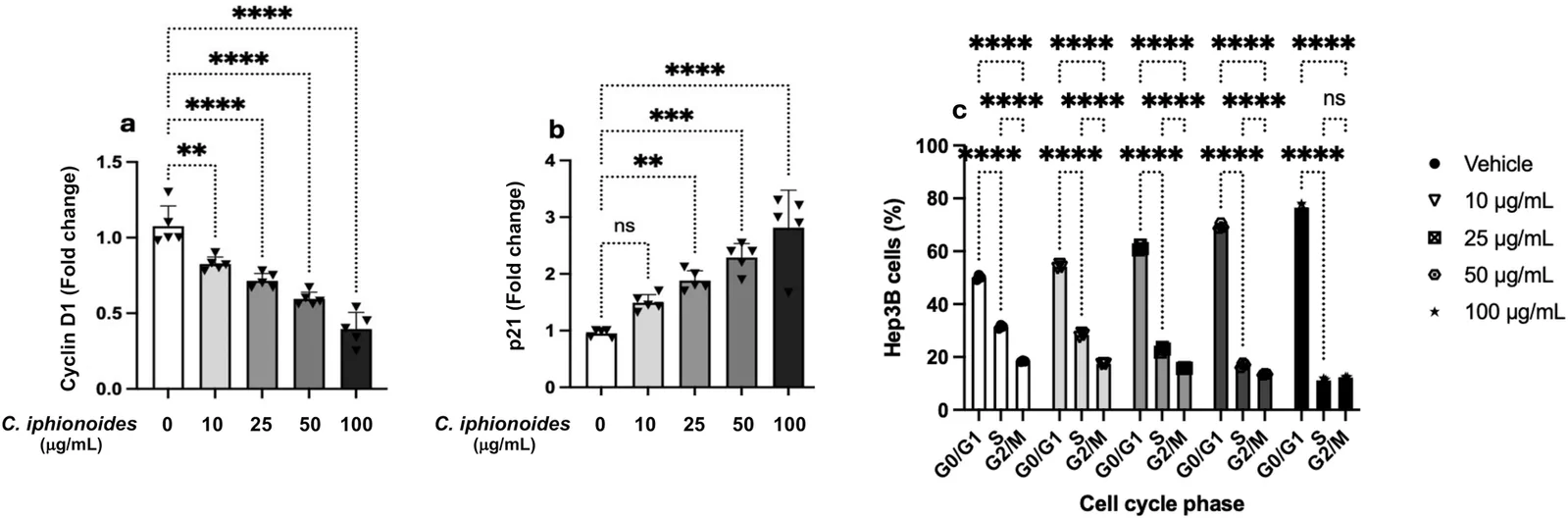
*C. iphionoides* ethanolic extract modulates cell-cycle-associated gene expression and induces G0/G1 arrest in Hep3B cells. Hep3B cells were treated with increasing concentrations of the ethanolic extract (10, 25, 50, and 100 µg/mL) or vehicle control (0.1% DMSO) for 24 h. Relative mRNA expression levels of **(a)** *cyclin D1* and **(b)** *p21* were determined by RT-qPCR, normalized to *GAPDH*, and expressed as the fold change relative to the vehicle-treated control group. **(c)** Cell-cycle distribution was determined by propidium iodide DNA content flow cytometry and is expressed as the percentage of gated cycling cells in G0/G1, S, and G2/M phases; sub-G1 events were excluded. Data in (a) and (b) represent the mean ± SD of five technical replicates, and data in (c) represent the mean ± SD of three replicates per group. Because the three phase proportions are compositional and sum to 100% within each sample, the treatment effects in (c) were evaluated within each phase separately rather than by interpreting an overall treatment main effect. Statistical significance was determined using one-way ANOVA followed by Tukey's multiple comparisons test. Asterisks indicate comparisons with the vehicle-treated control group: **p < 0.01, ***p < 0.001, ****p < 0.0001; ns, not significant.

Because oxidative stress and DNA damage can modulate cell-cycle progression through p53-independent pathways (29–31), the mRNA expression of key G1/S regulatory genes was evaluated by RT-qPCR following treatment with the *C. iphionoides* ethanolic extract. Relative cyclin D1 mRNA expression in untreated Hep3B cells was 1.08 ± 0.13 and decreased progressively to 0.83 ± 0.05, 0.71 ± 0.05, 0.59 ± 0.05, and 0.40 ± 0.11 following treatment with 10, 25, 50, and 100 µg/mL extract, respectively, corresponding to reductions of approximately 23.2%, 33.6%, 44.8%, and 63.2% relative to untreated controls (Figure 4a). In contrast, p21 mRNA expression increased markedly in a concentration-dependent manner. Relative p21 mRNA expression in untreated cells was 0.95 ± 0.06 and increased to 1.49 ± 0.14, 1.88 ± 0.17, 2.29 ± 0.25, and 2.82 ± 0.66 following treatment with 10, 25, 50, and 100 µg/mL extract, respectively, corresponding to increases of approximately 57.4%, 98.3%, 141.4%, and 197.1% relative to untreated controls (Figure 4b).

Notably, Hep3B is a TP53-null cell line where the TP53 gene is partially deleted due to hepatitis B virus genome integration, so that no p53 protein is expressed (32). Therefore, the observed induction of p21 mRNA reflects a p53-independent stress-response pathway rather than canonical p53-mediated transactivation. The reciprocal transcriptional regulation of cyclin D1 and p21 is consistent with the suppression of proliferative signaling in response to *C. iphionoides* treatment. As described below, these transcriptional changes were accompanied by corresponding shifts in cell-cycle distribution. However, p21 protein abundance was not quantified; therefore, the transcript data alone do not establish a change in p21 protein levels. Collectively, these findings indicate that the *C. iphionoides* ethanolic extract arrests Hep3B cells in G0/G1 and modulates the expression of cell-cycle-associated regulatory genes through a ROS-associated, p53-independent mechanism.

To determine whether these transcriptional changes were accompanied by a corresponding change in cell-cycle distribution, DNA content was analyzed by propidium iodide staining and flow cytometry. Treatment with *C. iphionoides* resulted in a concentration-dependent accumulation of cells in the G0/G1 phase, increasing from 50.10 ± 0.90% in vehicle-treated cells to 54.23 ± 0.78%, 61.30 ± 0.98%, 69.53 ± 1.12%, and 76.50 ± 1.42% following treatment with 10, 25, 50, and 100 µg/mL extract, respectively (*p* < 0.0001 vs. vehicle at each concentration) (Figure 4c). This accumulation was mirrored by a progressive depletion of the S-phase population, which decreased from 31.47 ± 0.65% in vehicle-treated cells to 28.33 ± 0.80% (*p* = 0.0004), 22.87 ± 0.91%, 16.97 ± 0.91%, and 11.23 ± 0.93% (*p* < 0.0001 for the three highest concentrations). The G2/M population decreased more modestly, from 18.43 ± 0.25% to 17.43 ± 0.15% (not significant), 15.83 ± 0.15% (*p* = 0.0035), 13.50 ± 0.30%, and 12.27 ± 0.49% (*p* < 0.0001 for the two highest concentrations). The reciprocal accumulation in G0/G1 and the loss of S-phase cells is the distribution expected from a block at the G1/S transition, providing functional confirmation of the reciprocal changes in the cyclin D1 and p21 transcript described above. Because sub-G1 events were excluded during gating, these proportions describe the cycling population and are not confounded by the apoptotic fraction quantified separately by Annexin V/PI staining.

### NAC attenuates *C. iphionoides*-induced ROS-mediated DNA damage and mitochondrial apoptosis in Hep3B cells

To determine whether ROS generation is mechanistically involved in *C. iphionoides*-induced cytotoxicity, Hep3B cells were pretreated with the antioxidant NAC before exposure to the *C. iphionoides* ethanolic extract. Pretreatment with NAC markedly attenuated the oxidative DNA damage induced by the extract, as demonstrated by significant reductions in intracellular 8-OHdG levels and *γ*H2AX expression compared with cells treated with *C. iphionoides* alone.

**Figure 5:**
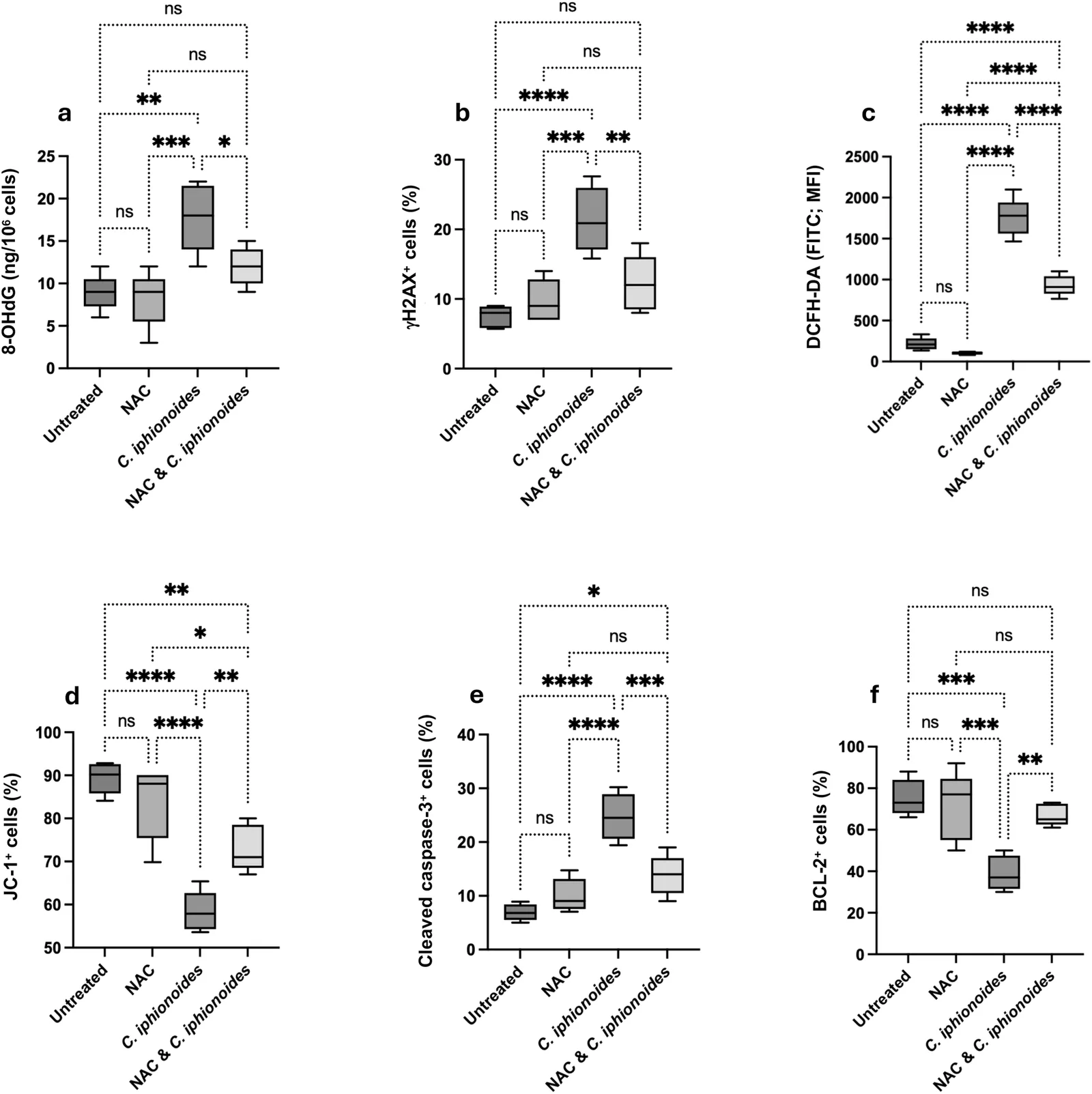
N-acetyl-L-cysteine attenuates *C. iphionoides*-induced oxidative stress, mitochondrial dysfunction, and apoptotic signaling in Hep3B cells. Hep3B cells were pretreated with 5 mM N-acetyl-L-cysteine (NAC) for 1 h and subsequently exposed to *C. iphionoides* ethanolic extract (50 µg/mL) for 24 h in the presence of NAC. Four groups were compared in each panel: untreated control, NAC alone, *C. iphionoides* alone, and NAC plus *C. iphionoides*. **(a)** Intracellular 8-hydroxy-2′-deoxyguanosine (8-OHdG) levels were quantified by competitive ELISA and expressed as ng per 10⁶ viable cells. **(b)** *γ*H2AX-positive cells were quantified by flow cytometry and expressed as the percentage of gated cells. **(c)** Intracellular reactive oxygen species were assessed using the 2′,7′-dichlorodihydrofluorescein diacetate (DCFH-DA) assay, with DCF fluorescence acquired in the FITC channel, and are expressed as the mean fluorescence intensity (MFI). **(d)** Mitochondrial membrane potential was evaluated using the JC-1 assay and expressed as the percentage of JC-1 aggregate-positive cells. **(e)** Cleaved caspase-3 and **(f)** BCL-2 expression were analyzed by flow cytometry and expressed as the percentage of positive cells. Data are shown as box plots, in which the horizontal line denotes the median, the box denotes the interquartile range, and the whiskers denote the minimum and maximum values; *n* = 5 technical replicates per group. Because these experiments involved two independent factors (extract treatment and NAC pretreatment), the statistical significance was determined by two-way ANOVA followed by Šídák's multiple comparisons test. Brackets denote the specific pairwise comparisons indicated: *p < 0.05, **p < 0.01, ***p < 0.001, ****p < 0.0001, ns; not significant.

Treatment with 50 µg/mL *C. iphionoides* extract increased intracellular 8-OHdG levels from 8.2 ± 2.30 ng/10⁶ cells in untreated controls to 18.00 ± 3 ng/10⁶ cells, whereas NAC pretreatment reduced 8-OHdG levels to 13.20 ± 2.63 ng/10⁶ cells (*p* < 0.05) (Figure 5a). Similarly, *γ*H2AX-positive Hep3B cells increased from 7.8 ± 2.4% in untreated controls to 21.5 ± 6.2% following treatment with *C. iphionoides* extract alone, while NAC pretreatment reduced *γ*H2AX positivity to 13.6 ± 4.5% (*p* < 0.01) (Figure 5b). Consistent with these findings, intracellular ROS production, assessed by the DCFH-DA assay, was markedly elevated following treatment with *C. iphionoides*, increasing DCF fluorescence from 216.0 ± 84.7 MFI in untreated cells to 1,756.2 ± 231.2 MFI. Pretreatment with NAC significantly attenuated ROS accumulation, reducing DCF fluorescence to 929.2 ± 122.8 MFI (*p* < 0.0001 vs. *C. iphionoides* alone) (Figure 5c). These findings confirm that ROS generation is an early upstream event that contributes to *C. iphionoides*-induced oxidative DNA damage and genotoxic stress in Hep3B cells.

Because oxidative DNA damage is associated with mitochondrial dysfunction and activation of intrinsic apoptotic signaling, the effects of NAC on the mitochondrial membrane potential and apoptotic markers were further evaluated. Treatment with *C. iphionoides* extract alone reduced JC-1 aggregate-positive cells from 89.4 ± 4.6% in untreated controls to 56.7 ± 5%, whereas NAC pretreatment significantly restored mitochondrial polarization to 71.8 ± 8.0% (*p* < 0.01) (Figure 5d), indicating partial preservation of mitochondrial integrity. In parallel, cleaved caspase-3-positive Hep3B cells increased from 7.9 ± 1.5% in untreated controls to 25.9 ± 4.7% following treatment with *C. iphionoides* extract alone, while NAC pretreatment reduced cleaved caspase-3 expression to 12.7 ± 4.1% (*p* < 0.001) (Figure 5e). Consistently, BCL-2-positive cells decreased from 72.2 ± 6.1% in untreated controls to 38.2 ± 4.7% following treatment with the extract alone, whereas NAC pretreatment partially restored BCL-2 expression to 63 ± 2.8% (*p* < 0.01) (Figure 5f). The NAC experiments were conducted as independent biological experiments performed separately from the dose–response studies. Therefore, the extract-alone values presented in Figure 5 represent the matched control group generated within the NAC experimental series and may differ slightly from the corresponding values obtained in the dose–response experiments. Despite minor interexperimental variability, the overall biological responses and treatment trends were highly consistent across experiments. Collectively, these findings demonstrate that ROS generation is a critical upstream event mediating *C. iphionoides*-induced oxidative DNA damage, mitochondrial dysfunction, and apoptotic cell death in Hep3B cells.

### *C. iphionoides* shows comparable activity in HepG2 and Huh-7 cells and limited activity in non-tumorigenic THLE-3 hepatocytes

To determine whether the responses observed in Hep3B cells were reproducible in other hepatocellular carcinoma models and whether they were selective for malignant hepatocytes, HepG2, Huh-7, and non-tumorigenic THLE-3 cells were treated with *C. iphionoides* ethanolic extract (50 μg/mL) or vehicle control for 24 h. Treatment significantly reduced *MKI67* mRNA expression in both HCC lines, from 1.00 ± 0.00 to 0.70 ± 0.03 in HepG2 cells (29.7% reduction) and from 0.96 ± 0.13 to 0.55 ± 0.10 in Huh-7 cells (42.5% reduction), whereas THLE-3 cells showed only a modest change (1.03 ± 0.15 vs. 0.85 ± 0.06; 17.7% reduction) (Supplementary Figure S3A). A similar pattern was observed for *VEGF-A*, which was markedly suppressed in HepG2 cells (1.03 ± 0.06 vs. 0.57 ± 0.18; 45.2% reduction) and Huh-7 cells (0.97 ± 0.12 vs. 0.53 ± 0.09; 44.8% reduction) but essentially unchanged in THLE-3 cells (0.97 ± 0.06 vs. 0.96 ± 0.15) (Supplementary Figure S3B).

The same selectivity was evident in the downstream mechanistic readouts. *γ*H2AX levels increased substantially in HepG2 cells (12.33 ± 0.65 vs. 20.77 ± 2.80; 68.4% increase) and Huh-7 cells (11.00 ± 1.00 vs. 21.17 ± 4.59; 92.4% increase), but only slightly in THLE-3 cells (7.63 ± 1.01 vs. 8.50 ± 3.28; 11.4% increase) (Supplementary Figure S3C). Caspase-9 activity increased from 328.0 ± 119.5 to 619.3 ± 94.7 in THLE-3 cells (88.8% increase; *P* = ns), but the response was considerably greater in HepG2 cells (351.7 ± 56.5 to 960.7 ± 99.9; 173.2% increase) and Huh-7 cells (403.3 ± 57.7 to 1,626.7 ± 230.7; 303.3% increase) (Supplementary Figure S3D). The mitochondrial membrane potential was largely preserved in THLE-3 cells (64.33 ± 11.26 vs. 54.67 ± 14.57; 15.0% reduction), whereas a pronounced loss was observed in HepG2 cells (78.33 ± 7.37 vs. 43.00 ± 11.79; 45.1% reduction) and Huh-7 cells (75.33 ± 7.64 vs. 45.00 ± 5.29; 40.3% reduction) (Supplementary Figure S3E). Taken together, these data indicate that the anticancer effects first observed in Hep3B cells are reproducible across additional hepatocellular carcinoma cell lines and are substantially weaker in non-tumorigenic hepatocytes, supporting the preferential sensitivity of malignant hepatocytes to *C. iphionoides*. It should be noted that THLE-3 cells were not entirely unaffected, indicating preferential rather than absolute selectivity.

## Discussion

To the best of our knowledge, this is the first study to demonstrate that the antihepatocellular carcinoma activity of *C. iphionoides* ethanolic extract exerts *in vitro* through a multifaceted mechanism involving reduced cell viability, intracellular ROS accumulation, oxidative DNA damage, mitochondrial dysfunction, activation of intrinsic apoptotic signaling, and p53-independent modulation of cell-cycle-associated gene expression. Treatment with *C. iphionoides* resulted in marked inhibition of Hep3B viability, along with significant reductions in AFP, GPC-3, and *VEGF-A* expression, indicating suppression of both proliferative capacity and tumor-associated functional activity. The observed IC_50_ value of 35.2 µg/mL (95% CI 31.85–38.47 µg/mL) is within the range reported for other crude plant extracts in hepatocellular carcinoma models. However, it should be interpreted with caution, as it refers to an uncharacterized multicomponent preparation rather than a defined compound.

Excessive ROS generation is a recognized mechanism by which numerous phytochemicals exert selective anticancer activity by overwhelming the antioxidant defense system of tumor cells (33, 34). In the present study, treatment with *C. iphionoides* markedly increased intracellular 8-OHdG levels and accumulated *γ*H2AX, indicating oxidative DNA injury and activation of DNA damage signaling pathways. Because *γ*H2AX is a highly sensitive marker of DNA double-strand breaks (20–22), its concentration-dependent induction strongly supports the involvement of ROS-mediated genotoxic stress in response to treatment. Importantly, the parallel increase in both 8-OHdG and *γ*H2AX provides mechanistic evidence that oxidative stress is a central upstream event in *C. iphionoides*-induced cytotoxicity. These findings are consistent with previous reports demonstrating that plant-derived anticancer compounds induce oxidative DNA damage and apoptosis in hepatocellular carcinoma cells through ROS-dependent mechanisms (19, 20, 35–38).

Oxidative DNA damage is accompanied by profound mitochondrial dysfunction and activation of intrinsic apoptotic signaling. JC-1 analysis demonstrated marked mitochondrial membrane depolarization following treatment, indicating disruption of mitochondrial integrity, which is considered a hallmark of early intrinsic apoptosis. This mitochondrial dysfunction was associated with a significant induction of Bax, increased caspase-9 activity and activation of cleaved caspase-3, increased PARP-1 cleavage, and marked suppression of the antiapoptotic protein BCL-2. Collectively, these findings support the activation of the mitochondrial apoptotic cascade in response to *C. iphionoides* treatment. The coordinated increase in Bax and caspase activation, along with the suppression of BCL-2, strongly suggests that *C. iphionoides* shifts Hep3B cells toward a pro-apoptotic state through mitochondrial-mediated apoptotic signaling.

Importantly, antioxidant experiments using NAC provided further mechanistic confirmation that ROS-associated oxidative stress is likely a critical upstream driver of the observed anticancer effects. NAC pretreatment significantly attenuated oxidative DNA damage, reduced *γ*H2AX accumulation, preserved mitochondrial membrane potential, suppressed caspase-3 activation, and partially restored BCL-2 expression following *C. iphionoides* exposure. Importantly, NAC alone would not have been sufficient to establish a ROS-specific mechanism, as NAC acts not only as an antioxidant and glutathione precursor but may also exert effects unrelated to ROS production. Therefore, the present study combined antioxidant rescue with direct quantification of intracellular ROS by DCFH-DA, and the concordance of the two approaches provides considerably stronger mechanistic support than either approach alone. The partial reversal of these downstream events following antioxidant pretreatment supports a ROS-associated mechanism linking oxidative DNA injury to mitochondrial apoptotic activation, although the individual ROS species involved and their precise intracellular sources remain unclear. These findings significantly strengthen the mechanistic framework of the present study by demonstrating not only an association but also the functional involvement of ROS generation in mediating *C. iphionoides*-induced Hep3B cell death.

Another notable finding of the present study was the induction of p21 mRNA and the suppression of cyclin D1 mRNA despite the absence of functional p53 in Hep3B cells. Hep3B is a TP53-null cell line in which the TP53 gene is partially deleted following hepatitis B virus genome integration, so that no p53 protein is expressed. Therefore, the p53-independent nature of the response reported here is established by the model's genetic background rather than inferred from expression data alone, and any p21 induction in this context must proceed through p53-independent regulators such as p73, Sp1, or TGF-*β*/Smad signaling. Previous studies have demonstrated that oxidative stress and phytochemical-derived anticancer agents can induce p21 independently of p53 through ROS-associated and MAPK-related signaling pathways (29–31). The reciprocal transcriptional regulation of cyclin D1 and p21 observed here is therefore consistent with impaired proliferative signaling in the absence of canonical p53-mediated regulation. DNA content analysis confirmed that these transcriptional changes are accompanied by a concentration-dependent accumulation of cells in G0/G1 with a reciprocal loss of the S-phase population, the distribution expected from a block at the G1/S transition. However, p21 protein abundance was not measured; therefore, the causal contribution of p21 to the observed arrest remains to be demonstrated directly, for example, by p21 knockdown. These findings are particularly relevant because TP53 dysfunction is common in advanced hepatocellular carcinoma and contributes to therapeutic resistance.

The anticancer activity observed in the present study aligns with the increasing body of evidence supporting the therapeutic potential of medicinal plants and phytochemical-rich extracts in hepatocellular carcinoma. Previous investigations have demonstrated the anticancer and antioxidant activities of *C. iphionoides* in other cancer models, including melanoma, breast cancer, and HepG2 cells (14, 38). The present study extends this work by describing an ROS-dependent mechanistic pathway involving oxidative DNA damage, mitochondrial dysfunction, intrinsic apoptosis, and p53-independent regulation of cell-cycle-associated genes in Hep3B cells. It also shows that the same responses are reproduced in HepG2 and Huh-7 cells, while being substantially attenuated in non-tumorigenic THLE-3 hepatocytes. This differential responsiveness suggests preferential activity against malignant hepatocytes, although THLE-3 cells were not completely unaffected, and a formal selectivity index across the full concentration range was not determined.

Collectively, the present findings support a mechanistic model in which the *C. iphionoides* ethanolic extract induces ROS-associated oxidative stress, leading to oxidative DNA damage, mitochondrial depolarization, activation of intrinsic apoptotic signaling, and suppression of proliferative pathways in Hep3B cells. Given its multitargeted activity in p53-null hepatocellular carcinoma cells, *C. iphionoides* warrants further preclinical investigation as a source of bioactive compounds for this type of cancer. However, these conclusions are restricted to cultured cells; the present study provides mechanistic evidence *in vitro* and does not establish therapeutic efficacy. Future studies should focus on chemical characterization and bioactivity-guided fractionation of the extract, validation in appropriate *in vivo* models, pharmacokinetic and toxicity assessment, and exploration of potential synergistic interactions with currently available anticancer therapies.

This study has several limitations. First, although the findings were reproduced in three hepatocellular carcinoma cell lines and compared with non-tumorigenic hepatocytes, all experiments were performed *in vitro*; therefore, the biological effects and any therapeutic relevance of *C. iphionoides* require confirmation in appropriate *in vivo* models. Second, the study evaluated a crude ethanolic extract and did not chemically characterize its individual constituents; therefore, the observed effects cannot be attributed to any specific compound or class of compounds. Comprehensive chromatographic and structural characterization using HPLC or HPTLC, LC-MS/MS, and NMR, followed by bioactivity-guided fractionation and evaluation of isolated compounds, fractions, and defined combinations, will be required to determine whether the activity arises from a single dominant constituent, from additive effects, or from synergistic interactions. Third, all reported experiments were performed using a single extract batch; therefore, interbatch variability in phytochemical composition and biological activity was not formally assessed. Fourth, although DNA content analysis demonstrated a concentration-dependent G0/G1 arrest, p21 protein abundance was not quantified; therefore, the mechanistic link between the induction of p21 transcript and the arrest has not been established directly. Fifth, GAPDH was used as the sole reference gene for RT-qPCR. Because *C. iphionoides* has previously been reported to influence metabolic pathways, GAPDH expression stability cannot be assumed, and future work should confirm these results using additional reference genes, such as RPLP0 or HPRT1. Sixth, assay-specific positive controls were not included for the oxidative stress, mitochondrial depolarization, apoptosis, and DNA damage assays. Although multiple complementary assays consistently supported the proposed mechanism, established positive controls would provide additional methodological validation. Finally, NAC is a non-specific antioxidant, and although direct ROS quantification was performed, ROS-specific probes together with pharmacological or genetic targeting of individual ROS-generating pathways will be needed to define the precise species and sources involved. Future studies addressing these limitations are important for the further development of *C. iphionoides*-derived anticancer strategies.

## Data Availability

The original contributions presented in this study are included in the article/Supplementary Material, further inquiries can be directed to the corresponding author.

## Supplementary material

Supplementary Figure S1 Dose–response curve for Hep3B cell viability used to determine the IC_50_ values.

Supplementary Figure S2 Annexin V-FITC/PI analysis of apoptosis in Hep3B cells.

Supplementary Figure S3 Effects of *C iphionoides* on HepG2, Huh-7, and THLE-3 cells. Representative flow cytometry plots and complete gating strategies applied to all markers.
