# Supplementary material for: Chiliadenus iphionoides ethanolic extract suppresses hepatocellular carcinoma cell survival through ROS-associated mitochondrial dysfunction and intrinsic apoptotic signaling in p53-null Hep3B cells
Source: Front Med (Lausanne). 2026 Sep 10;13:1914802. doi: 10.3389/fmed.2026.1914802 (PMC13600844; doi:10.3389/fmed.2026.1914802)
Supplement: Supplementary file 1 [file Datasheet1.pdf]

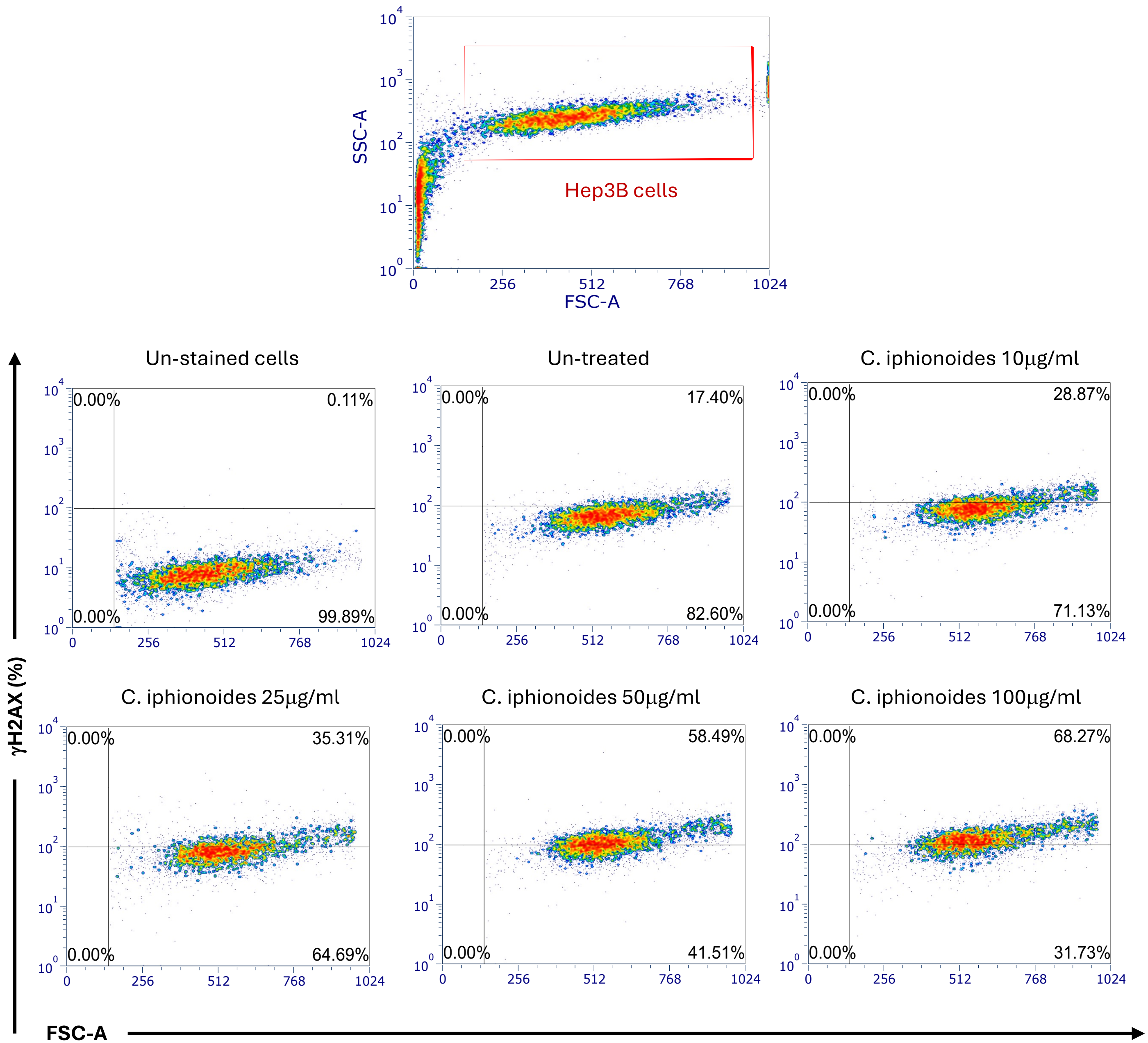
SSC-A
FSC-A
Hep3B cells
Un-stained cells
Un-treated
C. iphionoides 10µg/ml
C. iphionoides 25µg/ml
C. iphionoides 50µg/ml
C. iphionoides 100µg/ml
0.00% 0.11% 0.00% 99.89%
0.00% 17.40% 0.00% 82.60%
0.00% 28.87% 0.00% 71.13%
0.00% 35.31% 0.00% 64.69%
0.00% 58.49% 0.00% 41.51%
0.00% 68.27% 0.00% 31.73%
γH2AX (%)
FSC-A

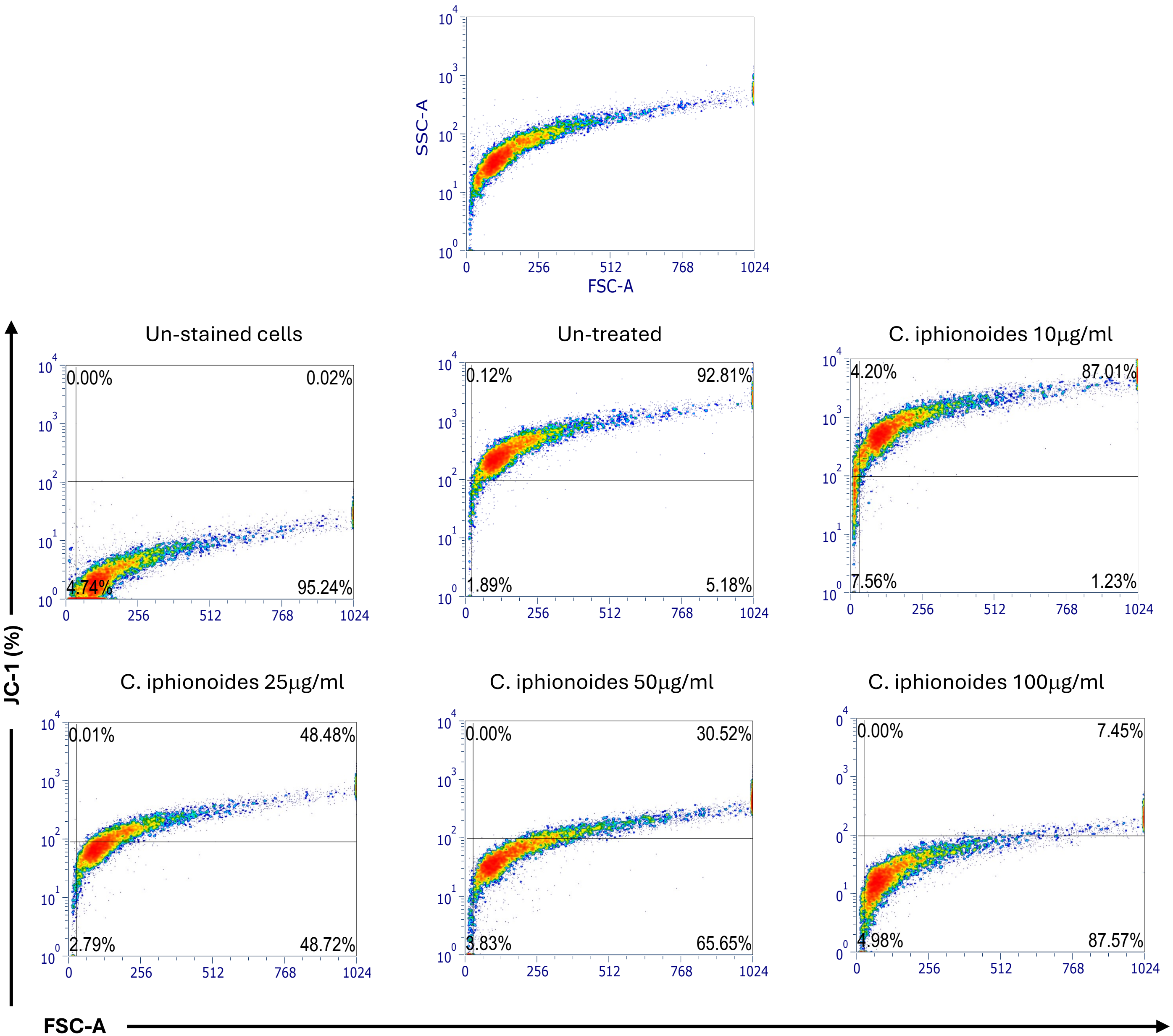
SSC-A
FSC-A
0
256
512
768
1024
Un-stained cells
0.00%
0.02%
4.74%
95.24%
Un-treated
0.12%
92.81%
1.89%
5.18%
C. iphionoides 10μg/ml
4.20%
87.01%
7.56%
1.23%
C. iphionoides 25μg/ml
0.01%
48.48%
2.79%
48.72%
C. iphionoides 50μg/ml
0.00%
30.52%
3.83%
65.65%
C. iphionoides 100μg/ml
0.00%
7.45%
4.98%
87.57%
JC-1 (%)
FSC-A

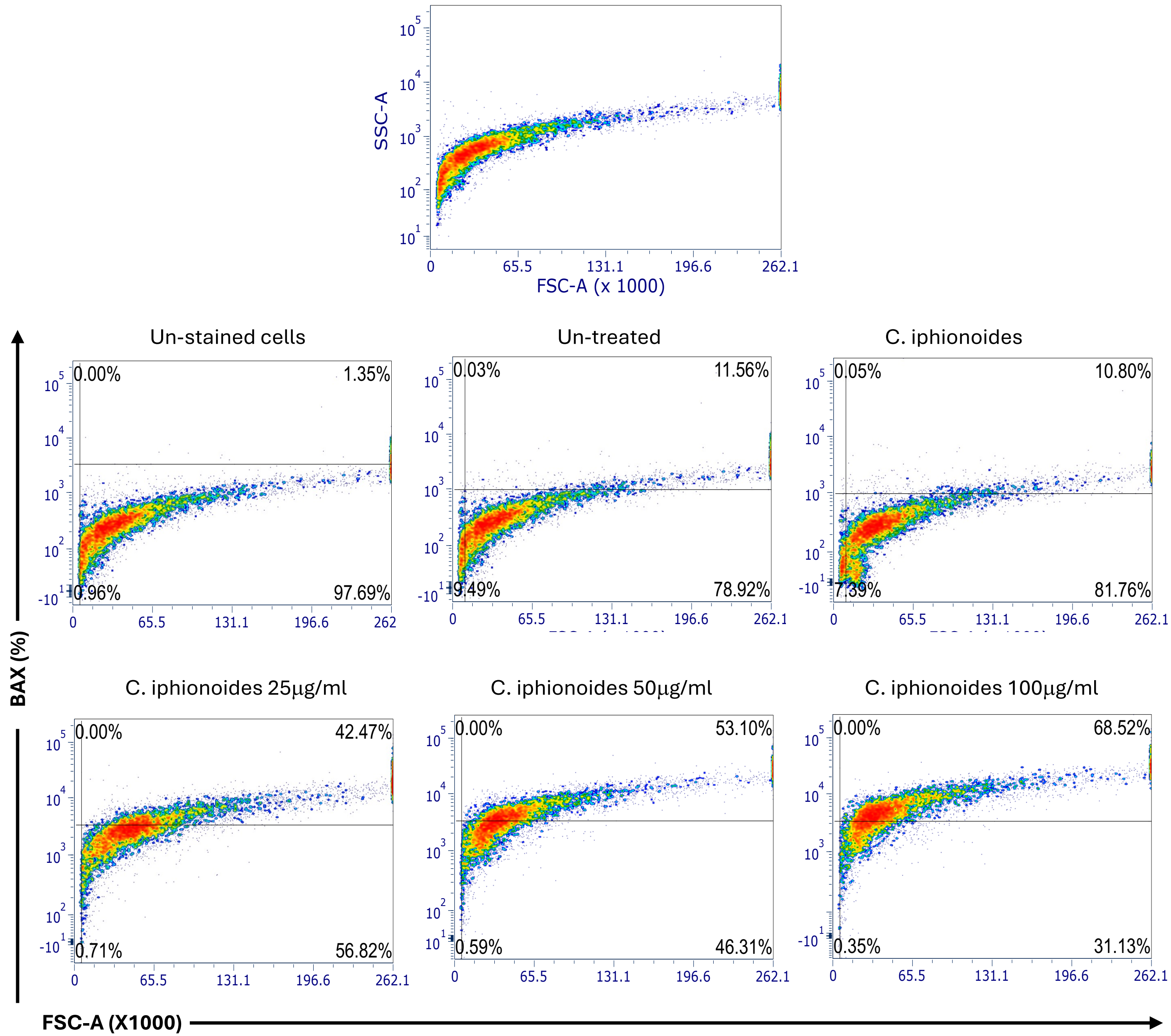
SSC-A
FSC-A (x 1000)
Un-stained cells
0.00%
1.35%
0.96%
97.69%
Un-treated
0.03%
11.56%
9.49%
78.92%
C. iphionoides
0.05%
10.80%
7.39%
81.76%
C. iphionoides 25µg/ml
0.00%
42.47%
0.71%
56.82%
C. iphionoides 50µg/ml
0.00%
53.10%
0.59%
46.31%
C. iphionoides 100µg/ml
0.00%
68.52%
0.35%
31.13%
BAX (%)
FSC-A (X1000)

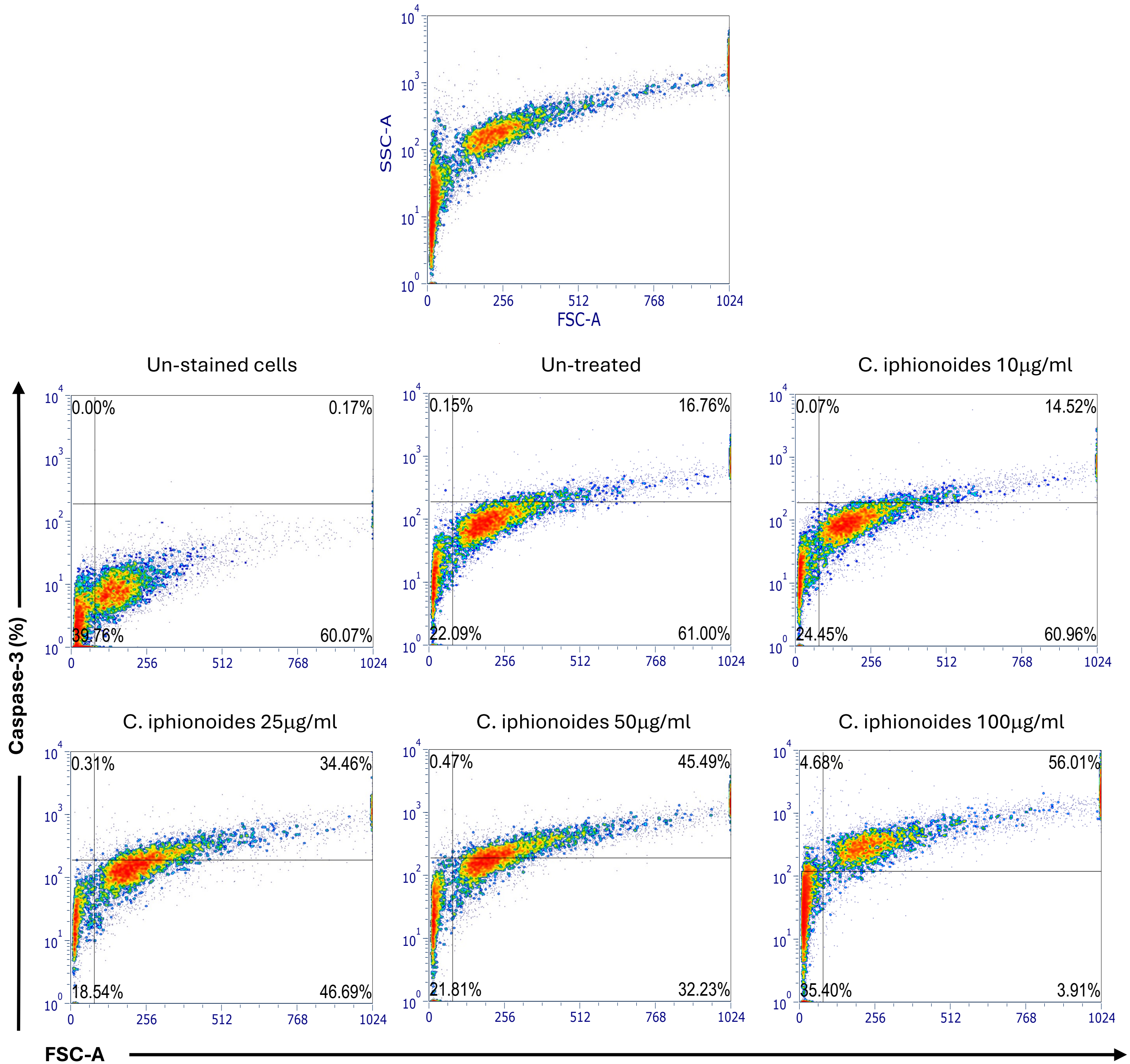
SSC-A
FSC-A
Un-stained cells
Un-treated
C. iphionoides 10µg/ml
C. iphionoides 25µg/ml
C. iphionoides 50µg/ml
C. iphionoides 100µg/ml
0.00%
0.17%
39.76%
60.07%
0.15%
16.76%
22.09%
61.00%
0.07%
14.52%
24.45%
60.96%
0.31%
34.46%
18.54%
46.69%
0.47%
45.49%
21.81%
32.23%
4.68%
56.01%
35.40%
3.91%
Caspase-3 (%)
FSC-A

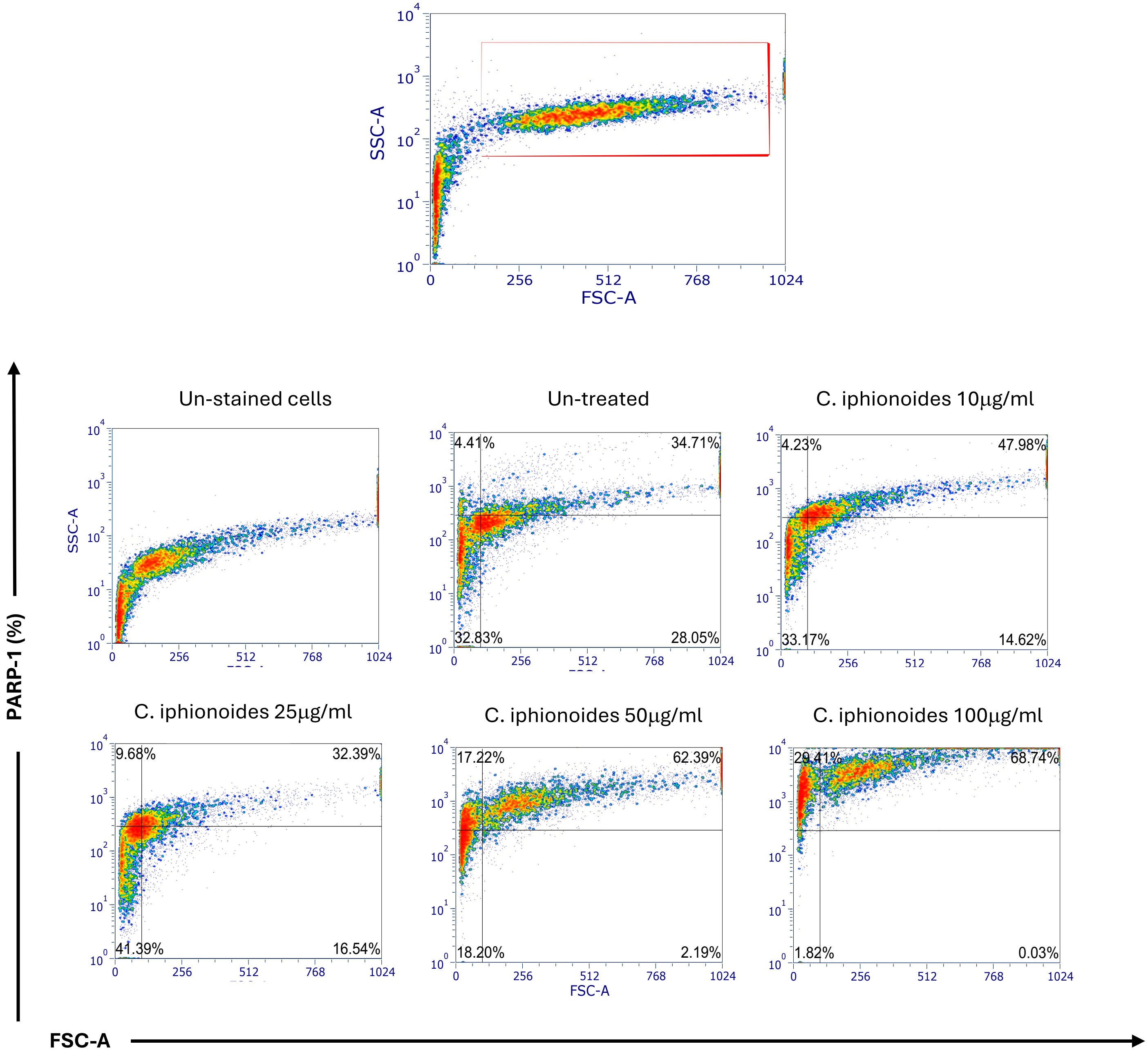

SSC-A
FSC-A
Un-stained cells
Un-treated
C. iphionoides 10µg/ml
C. iphionoides 25µg/ml
C. iphionoides 50µg/ml
C. iphionoides 100µg/ml
4.41%
34.71%
32.83%
28.05%
4.23%
47.98%
33.17%
14.62%
9.68%
32.39%
41.39%
16.54%
17.22%
62.39%
18.20%
2.19%
29.41%
68.74%
1.82%
0.03%
PARP-1 (%)
FSC-A

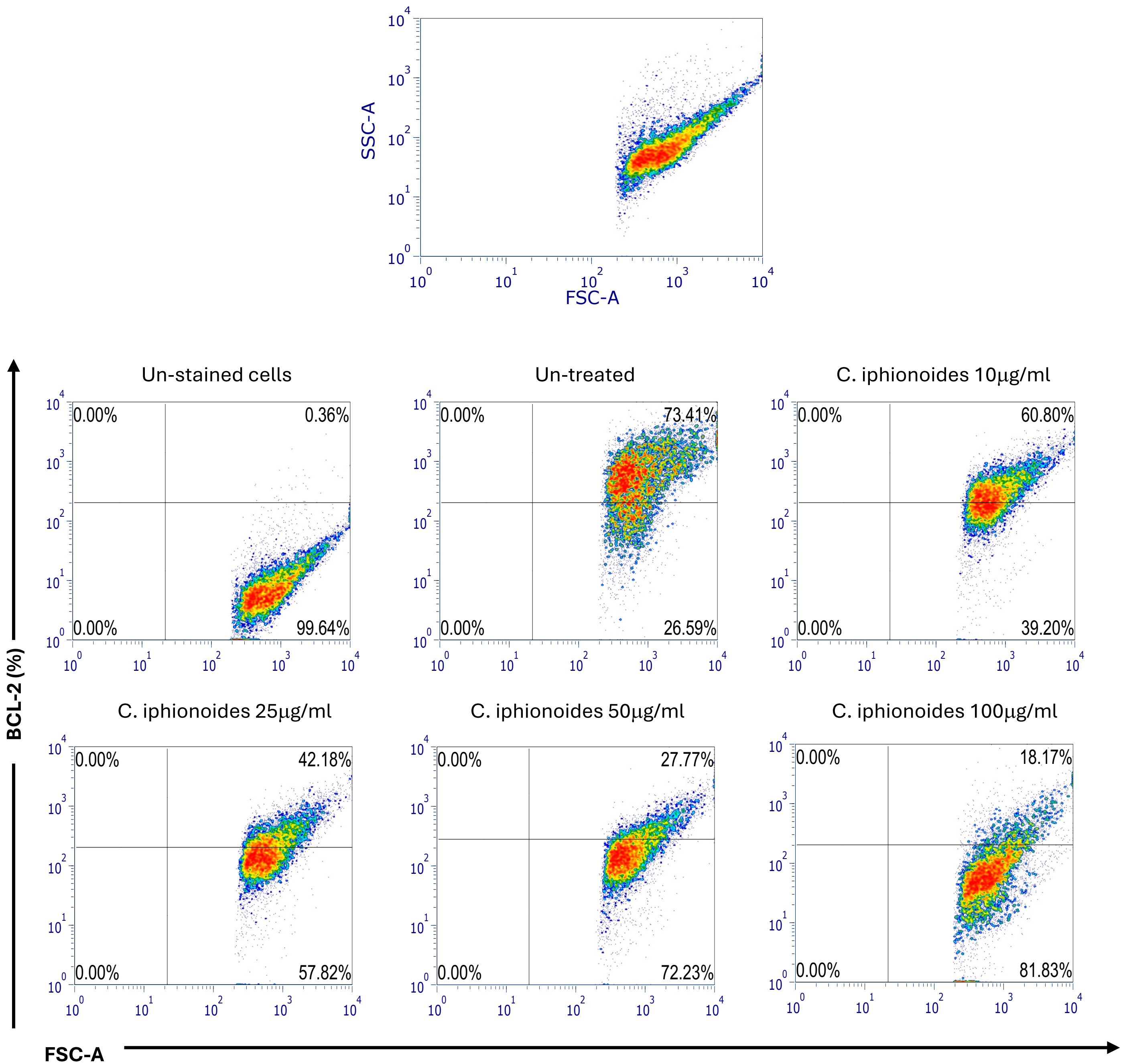
SSC-A
FSC-A
Un-stained cells
Un-treated
C. iphionoides 10µg/ml
C. iphionoides 25µg/ml
C. iphionoides 50µg/ml
C. iphionoides 100µg/ml
0.00%
0.36%
99.64%
73.41%
26.59%
60.80%
39.20%
42.18%
57.82%
27.77%
72.23%
18.17%
81.83%
BCL-2 (%)
FSC-A

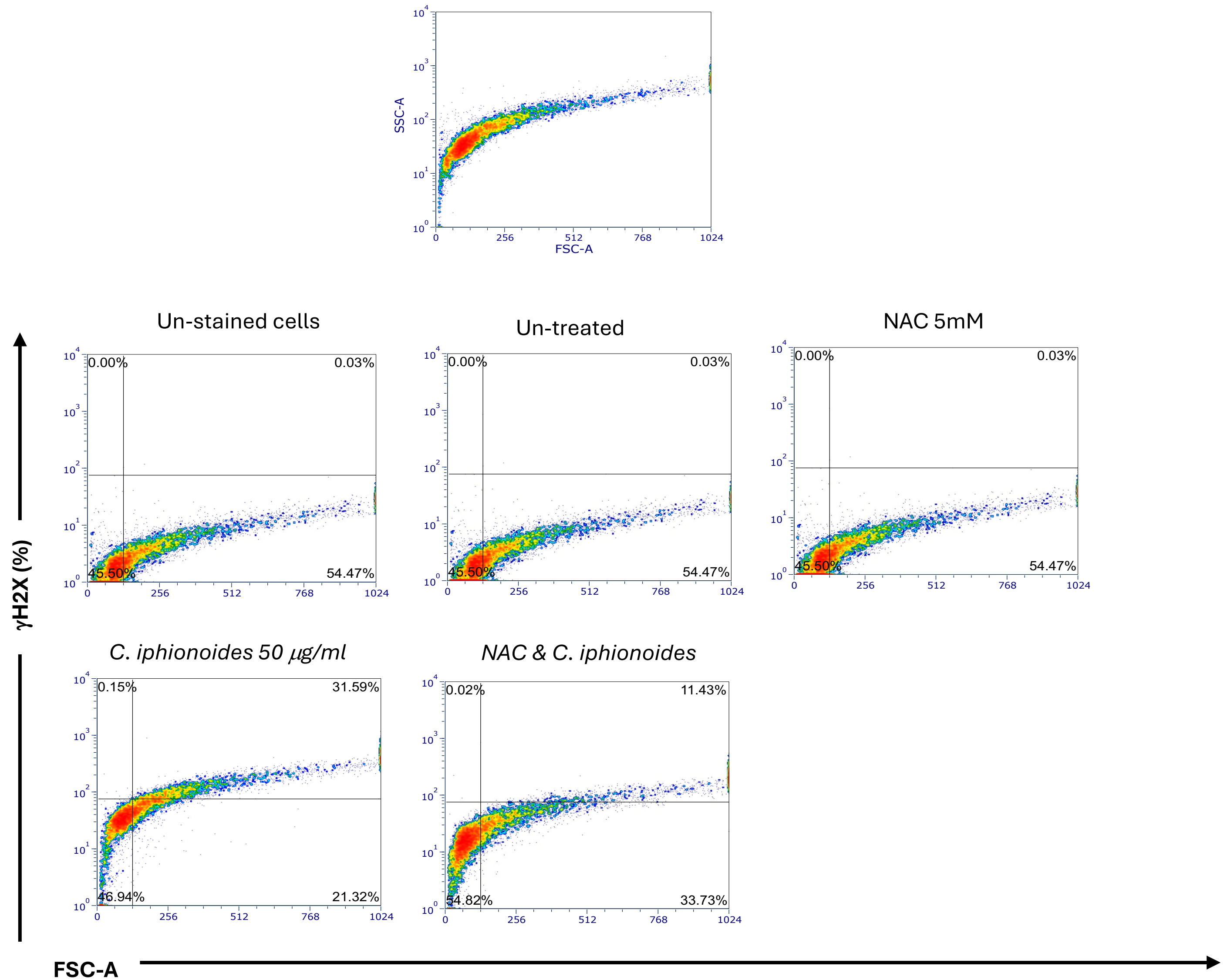
SSC-A
FSC-A
Un-stained cells
Un-treated
NAC 5mM
C. iphionoides 50 µg/ml
NAC & C. iphionoides
0.00%
0.03%
45.50%
54.47%
0.15%
31.59%
46.94%
21.32%
0.02%
11.43%
54.82%
33.73%
γH2X (%)
FSC-A

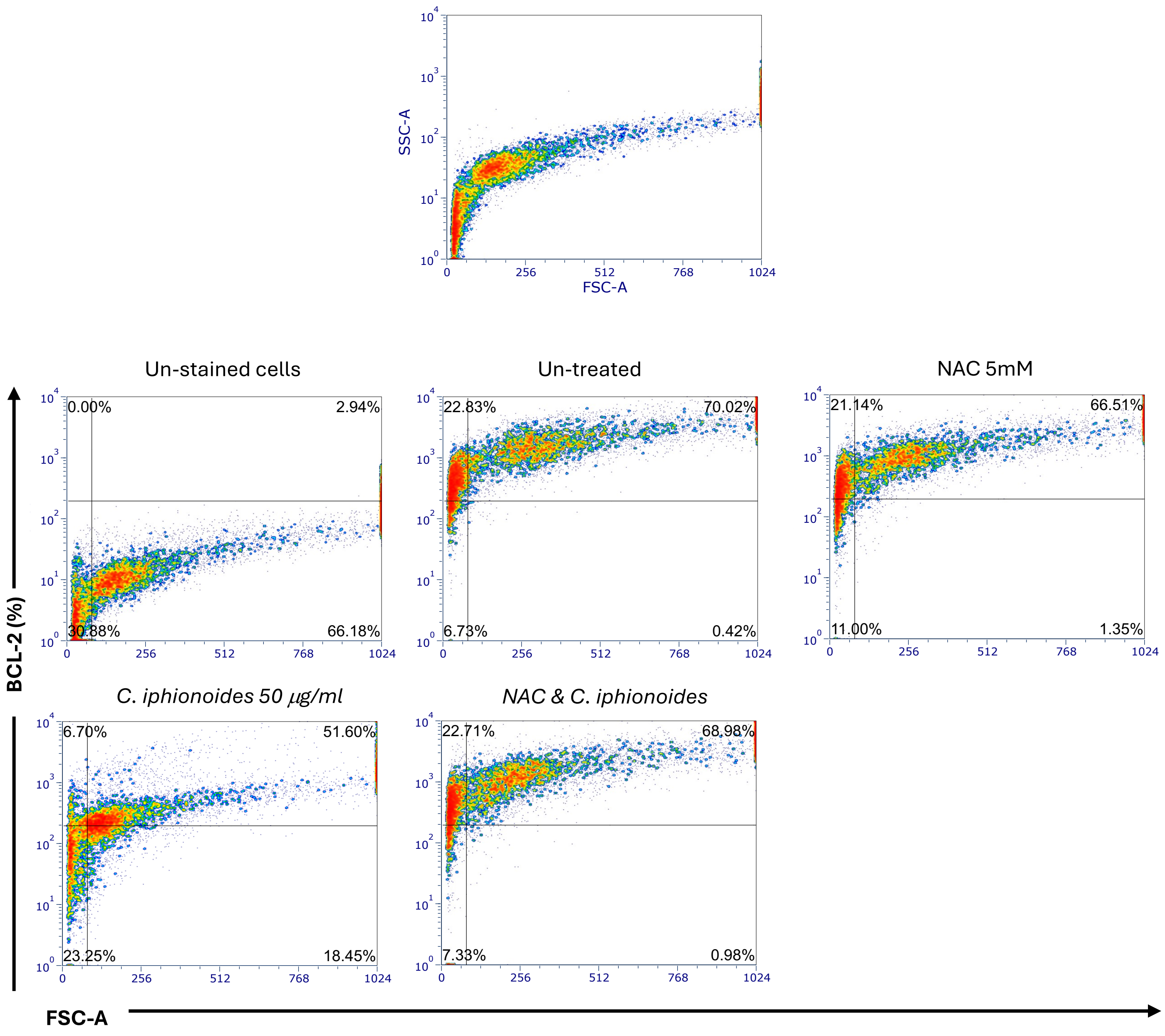

SSC-A
FSC-A
Un-stained cells
0.00%
2.94%
30.88%
66.18%
Un-treated
22.83%
70.02%
6.73%
0.42%
NAC 5mM
21.14%
66.51%
11.00%
1.35%
C. iphionoides 50 µg/ml
6.70%
51.60%
23.25%
18.45%
NAC & C. iphionoides
22.71%
68.98%
7.33%
0.98%
BCL-2 (%)
FSC-A
