# Supplementary material for: Chiliadenus iphionoides ethanolic extract suppresses hepatocellular carcinoma cell survival through ROS-associated mitochondrial dysfunction and intrinsic apoptotic signaling in p53-null Hep3B cells
Source: Front Med (Lausanne). 2026 Sep 10;13:1914802. doi: 10.3389/fmed.2026.1914802 (PMC13600844; doi:10.3389/fmed.2026.1914802)
Supplement: Supplementary file 2 [file Table1.docx]

**Supplementary Table S1. Individual technical replicate values underlying the Hep3B cell viability data presented in Table 1.**

| **Concentration** **(µg/mL)** | **Replicate 1** | **Replicate 2** | **Replicate 3** | **Replicate 4** | **Replicate 5** | **Mean ± SD** |
| --- | --- | --- | --- | --- | --- | --- |
| 0 (vehicle) | 96.84 | 98.42 | 100.00 | 101.58 | 103.16 | **100.0 ± 2.5** |
| 10 | 88.31 | 90.20 | 92.10 | 94.00 | 95.89 | **92.1 ± 3.0** |
| 25 | 63.56 | 65.33 | 67.10 | 68.87 | 70.64 | **67.1 ± 2.8** |
| 50 | 33.14 | 34.47 | 35.80 | 37.13 | 38.46 | **35.8 ± 2.1** |
| 100 | 12.30 | 13.50 | 14.70 | 15.90 | 17.10 | **14.7 ± 1.9** |

Values are percent cell viability relative to vehicle-treated controls, determined by the MTS assay after 24 h of treatment with *C. iphionoides* ethanolic extract, following background correction against both medium-only and extract-only blank wells (medium + extract + MTS reagent, without cells). Five technical replicates were measured per concentration within the experiment. The mean ± SD values in the final column correspond to those summarized in Table 1 of the main article, and these data were used for the nonlinear regression from which the IC_50_ was derived.

*Abbreviations:* IC_50_, half-maximal inhibitory concentration; MTS, 3-(4,5-dimethylthiazol-2-yl)-5-(3-carboxymethoxyphenyl)-2-(4-sulfophenyl)-2H-tetrazolium; SD, standard deviation.
